# *Fis1* ablation in the male germline disrupts mitochondrial morphology and mitophagy, and arrests spermatid maturation

**DOI:** 10.1242/dev.199686

**Published:** 2021-08-12

**Authors:** Grigor Varuzhanyan, Mark S. Ladinsky, Shun-ichi Yamashita, Manabu Abe, Kenji Sakimura, Tomotake Kanki, David C. Chan

**Affiliations:** 1 Division of Biology and Biological Engineering, California Institute of Technology, Pasadena CA 91125, USA; 2 Department of Cellular Physiology, Niigata University Graduate School of Medical and Dental Sciences, Niigata 951-8510, Japan; 3 Department of Animal Model Development, Brain Research Institute, Niigata University, Niigata 951-8585, Japan

**Keywords:** Autophagy, Mitochondrial dynamics, Mitophagy, Spermatid, Spermatogenesis, Mouse

## Abstract

Male germline development involves choreographed changes to mitochondrial number, morphology and organization. Mitochondrial reorganization during spermatogenesis was recently shown to require mitochondrial fusion and fission. Mitophagy, the autophagic degradation of mitochondria, is another mechanism for controlling mitochondrial number and physiology, but its role during spermatogenesis is largely unknown. During post-meiotic spermatid development, restructuring of the mitochondrial network results in packing of mitochondria into a tight array in the sperm midpiece to fuel motility. Here, we show that disruption of mouse *Fis1* in the male germline results in early spermatid arrest that is associated with increased mitochondrial content. Mutant spermatids coalesce into multinucleated giant cells that accumulate mitochondria of aberrant ultrastructure and numerous mitophagic and autophagic intermediates, suggesting a defect in mitophagy*.* We conclude that *Fis1* regulates mitochondrial morphology and turnover to promote spermatid maturation.

## Abstract (precis)

**Summary:** Analysis of germ cell-specific *Fis1* knockout mice and mitophagy reporter mice reveals that the mitochondrial dynamics gene *Fis1* regulates mitochondrial morphology and turnover during spermatid maturation.

## INTRODUCTION

Male germline development (spermatogenesis) is one of biology's most complex developmental programs, transforming spermatogonial stem cells into highly specialized sperm cells capable of fertilization. Spermatogenesis requires successive cycles of germ cell differentiation within the seminiferous epithelium. This tightly controlled process is regulated by somatic Sertoli cells, which intercalate with the germ cells and control their microenvironment (Griswold, 2016). As spermatogonial stem cells differentiate into sperm cells (spermatozoa), they progressively migrate from the seminiferous tubule periphery towards the lumen. Spermatozoa are then released into the lumen and transported to the epididymides via ATP-dependent tubular contractions (Fleck et al., 2021). Spermatogenesis is initiated by a pulse of retinoic acid that travels like a wave along the length of a seminiferous tubule. As a result, different regions along the longitudinal axis of a tubule are in distinct developmental phases and display unique cellular associations, referred to as ‘stages’ (Griswold, 2016; Russell et al., 1993). The stages of the seminiferous epithelium are defined by the developmental ‘steps’ of post-meiotic spermatids. In mice, spermatid development is divided into 16 steps, which are defined by the spermatid morphology as well as the extent of acrosome maturation.

Spermatogenesis is generally divided into three broad categories: (1) mitotic amplification of spermatogonia before they differentiate into spermatocytes, (2) meiotic division of spermatocytes to form haploid spermatids, and (3) maturation of spermatids into spermatozoa – a process termed spermiogenesis. The unique physiological requirements of these different germ cell types are regulated by mitochondrial dynamics (fusion and fission) (Varuzhanyan and Chan, 2020). In undifferentiated spermatogonia, mitochondria are generally small and fragmented. As spermatogonia differentiate into spermatocytes and initiate meiosis, their mitochondria undergo mitofusin-mediated fusion to fuel meiosis (Chen et al., 2020; Varuzhanyan et al., 2019; Wang et al., 2021; Zhang et al., 2016). In post-meiotic spermatids, acute mitochondrial fragmentation mediated by mitochondrial fission factor (MFF) generates small mitochondrial spheres, which enables their organization into a spiral array within the midpiece (Varuzhanyan et al., 2021). At the end of spermatid maturation, excess cellular components, including mitochondria, are culled into residual bodies for heterophagic degradation in Sertoli cells (Chemes, 1986). Thus, dynamic restructuring of mitochondria takes place throughout spermatogenesis.

Although key roles for mitochondrial fusion and fission have been demonstrated in spermatogenesis (Varuzhanyan and Chan, 2020), the role of mitophagy – the autophagic degradation of mitochondria (Pickles et al., 2018) – is largely unknown (Lv et al., 2020; Rathje et al., 2019). Because mitophagy counterbalances mitochondrial biogenesis and can remove dysfunctional mitochondria, it can control mitochondrial abundance and quality. There is evidence that the autophagy pathway functions to eliminate excess cellular material in spermatids during their transformation into highly compacted sperm cells. Deletion of the core autophagy gene *Atg7* has been shown to diminish autophagic flux in spermatids, cause acrosome fragmentation (Wang et al., 2014), and prevent spermatid polarization and cytoplasmic removal (Shang et al., 2016). However, it remains unknown whether spermatids use mitophagy to control mitochondrial density and remodeling.

The mitochondrial dynamics gene *Fis1* has been shown to mediate mitochondrial fission in the budding yeast *Saccharomyces cerevisiae* (Fekkes et al., 2000; Kraus et al., 2021; Mozdy et al., 2000; Tieu and Nunnari, 2000). However, mammalian *Fis1* has little (Losón et al., 2013) or no (Osellame et al., 2016; Otera et al., 2010) role in mitochondrial fission. Instead, *Fis1* has been implicated in mitophagy in multiple species and in a variety of cell types, for example cultured cells (Rojansky et al., 2016; Shen et al., 2014; Xian et al., 2019; Yamano et al., 2014, 2018), nematodes (Shen et al., 2014), early mouse embryos (Rojansky et al., 2016), mouse skeletal muscles (Zhang et al., 2019), and leukemia stem cells (Pei et al., 2018). Furthermore, *Fis1* was recently implicated in an asymmetric type of mitochondrial fission that is associated with mitophagy (Kleele et al., 2021). Some evidence indicates that *Fis1* may also have a more general function during nonselective autophagy. *Fis1*-deficient worms treated with mitochondrial toxins accumulate large autophagic structures (Shen et al., 2014). Furthermore, *Fis1* can regulate mitochondrion-lysosome contacts via the *Tbc1d15/Rab7* pathway (Wong et al., 2018; Yu et al., 2020), and can affect lysosomal function (Joshi et al., 2019; Kim et al., 2016). Finally, *Fis1* genetically interacts with the amyotrophic lateral sclerosis gene *C9orf72* (Chai et al., 2020), which is involved in membrane trafficking and autophagy (Nassif et al., 2017). Thus, *Fis1* is implicated in mitophagy and may have a more general role in regulating nonselective autophagy.

Here, we investigate the role of *Fis1* during mouse spermatogenesis, a process that is highly sensitive to perturbations in mitochondrial dynamics and autophagy. To this end, we generated and characterized male germ cell-specific *Fis1* knockout mice and male germ cell-specific mitophagy reporter mice. Our analysis indicates that *Fis1* is required for the development of the male germline by regulating mitochondrial morphology, mitophagy and autophagy during spermatid maturation.

## RESULTS

### *Fis1* is required for spermatogenesis

To study the role of *Fis1* during male germline development, we generated mice with a conditional *Fis1* allele (Fig. S1A). To remove *Fis1* from the male germline, we crossed conditional *Fis1* mice to the *Stra8-Cre* driver (Sadate-Ngatchou et al., 2008). We refer to the mutants as S8/*Fis1*, and their littermate controls as S8/Control. *Stra8-Cre* expression begins at postnatal day (P) 3 (Sadate-Ngatchou et al., 2008) in the majority of stem-like GFRA1-positive spermatogonia (Hobbs et al., 2015; Varuzhanyan et al., 2019). Therefore, all male germ cell types should be depleted of *Fis1*. We confirmed gene knockout by genotype analysis of tail DNA (Fig. S1B) and immunostaining of testis sections with an antibody against FIS1 (Fig. S1C). In control mice, *Fis1* is expressed in the mitochondria of both germ and Sertoli cells. In mutant mice, *Fis1* expression is eliminated selectively from germ cells.

**Fig. 1.**
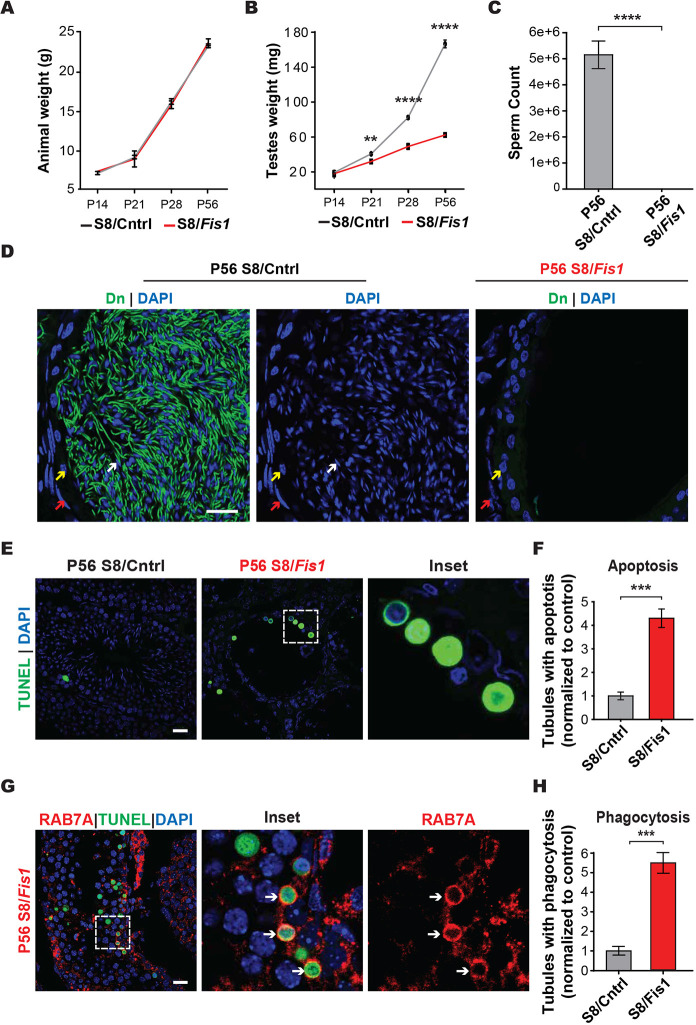
***Fis1* is required for spermatogenesis.** (A) Longitudinal analysis of animal weight. (B) Longitudinal analysis of testis weight. (C) Epididymal sperm count. Spermatozoa from both caudal epididymides were counted. (D) Histological analysis of caudal epididymis sections. Nuclei were stained with DAPI (blue), and mitochondria were labeled with mito-Dendra2 (Dn) (green). Note that no spermatozoa were present in the mutant sample. Red arrows, smooth muscle cells; yellow arrows, basal cells; white arrows; spermatozoa. Scale bar: 20 µm. (E) TUNEL staining (green) to detect apoptotic cells in testis sections. Scale bar: 20 µm. (F) Quantification of the number of tubules containing one or more apoptotic germ cells, normalized to control. (G) Histological analysis of apoptotic cells. Apoptotic cells (green) are surrounded by a RAB7A-positive structure (red), which is likely a Sertoli cell phagosome (white arrows). Scale bar: 20 µm. (H) Quantification of the number of tubules containing one or more RAB7A phagosomes, normalized to control. All data are from adult (P56) mice. Data are represented as mean±s.e.m. ****P≤0.0001; ***P≤0.001. For statistical tests used, see the Materials and Methods section.

S8/*Fis1* mice were healthy, displaying no changes in body weight compared with controls (Fig. 1A). However, their testes were smaller and weighed substantially less than those of age-matched controls (Fig. 1B). Mutant epididymides were completely devoid of spermatozoa (Fig. 1C,D), indicating an essential role for *Fis1* during spermatogenesis. To examine apoptosis, we performed terminal deoxynucleotidyl transferase dUTP nick end labeling (TUNEL) of testis sections. S8/*Fis1* testes had a greater than four-fold increase in TUNEL-positive tubules, indicating increased cell death by apoptosis (Fig. 1E,F). In cultured cells, downregulation of *Fis1* has been shown to inhibit apoptosis (Lee et al., 2004); therefore, these results indicate that the effect of *Fis1* depletion on apoptosis is highly context dependent.

A basal level of apoptosis occurs during normal germ cell development, and the affected germ cells are phagocytosed by Sertoli cells (Braun, 1998; Elliott et al., 2010). To address whether the large numbers of apoptotic germ cells in S8/*Fis1* mutants were similarly phagocytosed, we performed the TUNEL assay and co-stained for RAB7A, a small GTPase associated with phagophore maturation and subsequent fusion with lysosomes (Zhang et al., 2009) (Fig. 1G,H). In control testes, about one-third of TUNEL-positive cells were enclosed by ring-like structures decorated with RAB7A, suggesting that they are on the pathway to phagocytic degradation. S8/*Fis1* sections had a five-fold increase in the number of RAB7A-positive phagosomes. Thus, depletion of *Fis1* causes apoptotic loss of germ cells, which are then likely eliminated by Sertoli cell phagocytosis.

### Germ cell *Fis1* deletion results in multinucleated spermatid giant cells

**Fig. 2.**
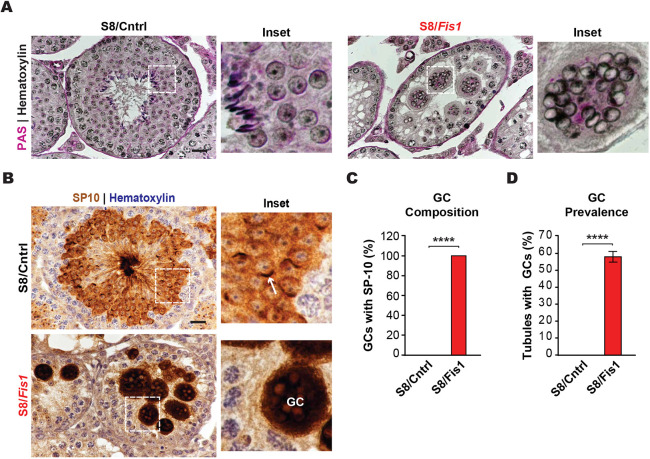
**Germ cell *Fis1* deletion results in multinucleated spermatid giant cells.** (A) PAS staining of adult testis sections, counterstained with Hematoxylin. Note the large, multinucleated GCs in S8/*Fis1* testes. Scale bar: 20 µm. (B) Immunohistochemical staining of testis sections with an antibody against the spermatid-specific SP-10 protein. The acrosome in a control round spermatid is indicated by a white arrow. Spermatids in mutant GCs lack acrosomes and stain intensely for SP-10. Scale bar: 20 µm. (C) Quantification of the number of GCs with SP-10 reactivity. (D) Quantification showing the prevalence of spermatid GCs in *Fis1* mutants. Control testes never exhibit GCs. All data are from adult (P56) mice. Data are represented as mean±s.e.m. ****P≤0.0001. For statistical tests used, see the Materials and Methods section.

To gain a better understanding of the spermatogenic defect in S8/*Fis1* mice, we performed periodic acid-Schiff (PAS) staining of adult testis sections (Fig. 2A). Control seminiferous tubules exhibited classical germ cell organization and their lumens were filled with spermatozoa. In stark contrast, S8/*Fis1* tubules were devoid of spermatozoa and were filled with structures that resemble previously described multinucleated giant cells (GCs) (MacGregor et al., 1990). To verify that these structures are multinucleated, we stained testis sections with DAPI and visualized germ cell boundaries with the plasma membrane marker sodium/potassium-transporting ATPase subunit alpha-1 (Na/K-ATPase) (Fig. S2). GCs did indeed contain multiple nuclei that were not compartmentalized by plasma membrane. The nuclear morphology of the GCs indicated that they are comprised primarily of spermatids. To verify this, we immunostained testis sections with the spermatid-specific acrosome marker SP-10 (Acrv1) (Osuru et al., 2014). In control testis sections, SP-10 expression was restricted to round and elongating spermatids, with the most intense staining highlighting the crescent-shaped acrosome (Fig. 2B). In S8/*Fis1* sections, GCs stained intensely for SP-10, which was present diffusely throughout the GC cytosol (Fig. 2B,C). Finally, the majority of S8/*Fis1* tubules contained GCs (Fig. 2D), indicating that they are a prominent pathological feature in mutant testes.

### *Fis1* giant cells have ectopic γH2AX expression

**Fig. 3.**
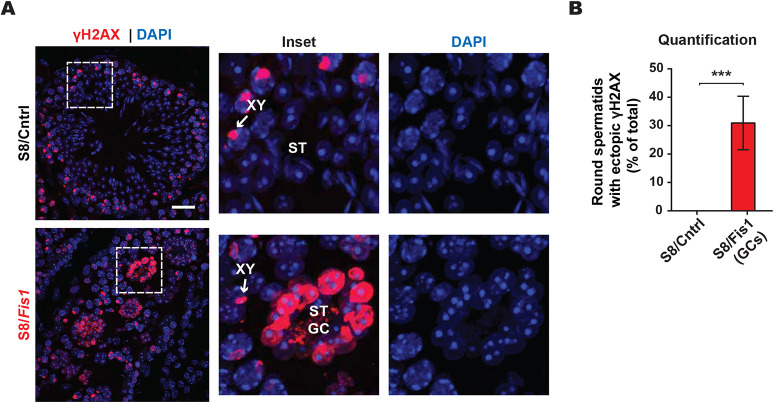
**S8/*Fis1* giant cells have ectopic γH2AX expression.** (A) Immunostaining in P35 testis sections with an antibody against the DSB repair protein γH2AX. The XY bodies are indicated by white arrows. Note the robust, ectopic expression of γH2AX in the *Fis1* spermatid GCs. ST, spermatid; ST GC; spermatid giant cell. Scale bar: 20 µm. (B) Quantification showing the percentage of round spermatids with γH2AX staining. In the mutant, quantification shows the percent of spermatid GCs that contain one or more cells with ectopic γH2AX expression. Data are represented as mean±s.e.m. ***P≤0.001. For statistical tests used, see the Materials and Methods section.

Because S8/*Fis1* mice exhibit arrest in spermatid development, we next checked whether spermatid precursor cells, the meiotic spermatocytes, displayed any abnormalities. We visualized spermatocytes by staining testis sections with the double-strand break (DSB) repair protein γH2AX, which differentially labels spermatocytes in different stages of meiosis. γH2AX is first observed in early prophase I spermatocytes, when programmed DSBs are generated to enable homologous recombination (Hamer et al., 2003; Mah et al., 2010). During pachytene, these DSBs are resolved as homologous recombination takes place, but γH2AX persists on the XY body, which is thought to be associated with silencing of the unsynapsed sex chromosomes (Baarends et al., 2005; Fernandez-Capetillo et al., 2003; Turner, 2007). Our analysis indicated that pachytene spermatocytes formed normally in S8/*Fis1* mice (Fig. 3A).

By the end of meiosis I, the γH2AX signal is completely resolved and does not reappear until the histone to protamine transition in steps 10-12 spermatids (Jha et al., 2017; Meistrich et al., 2003). Consistent with these epigenome dynamics, control round spermatids lacked γH2AX staining (Fig. 3A,B). Unexpectedly, we found that 30% of S8/*Fis1* GCs have ectopic γH2AX expression. We checked whether cells with ectopic γH2AX expression are apoptotic. However, TUNEL staining showed that γH2AX-positive cells in mutant samples were not associated with TUNEL labeling (Fig. S3).

### *Fis1* is required for acrosome homoeostasis

**Fig. 4.**
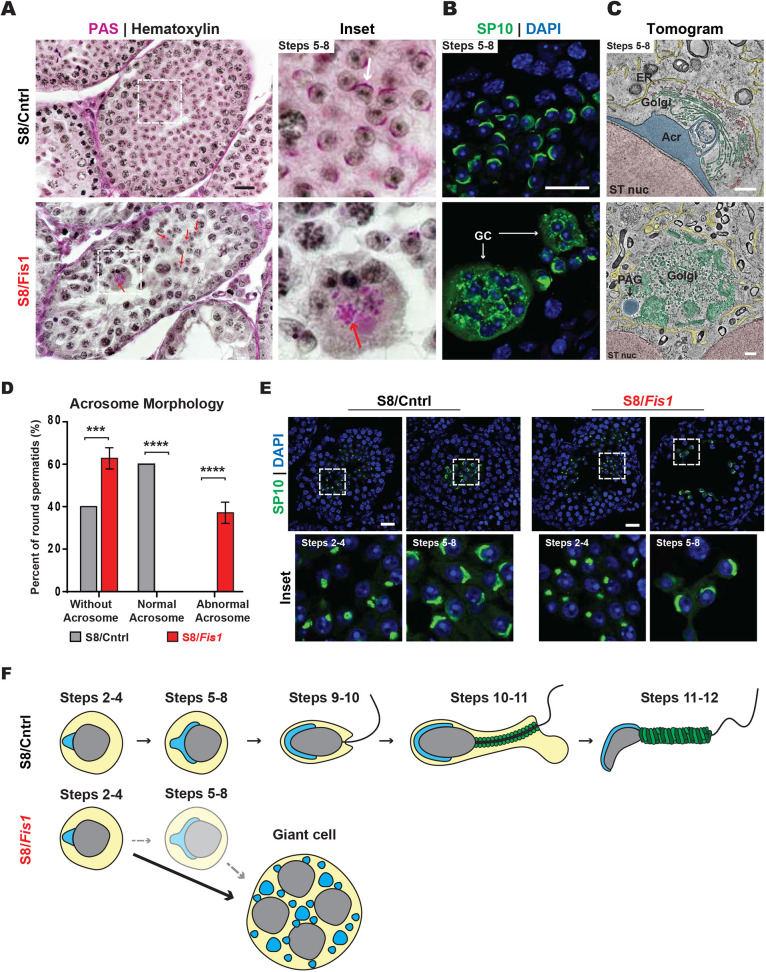
***Fis1* is required for acrosome homeostasis.** (A) PAS staining of P28 testis sections. The steps of spermatid development are indicated. In the control sample (top), each acrosome appears as a purple crescent, juxtaposed to the spermatid nucleus. A normal acrosome in control is marked by a white arrow and fragmented acrosomes in the mutant are indicated by red arrows. Scale bar: 20 µm. (B) SP-10 immunostaining in P28 testis sections. The differentiation step in mutant spermatids could not be precisely determined because the aberrant acrosomal structures do not correspond to a normal differentiation step. However, the SP10-positive structures in GCs resemble those of spermatids in steps 2-4. Scale bar: 20 µm. (C) Electron tomography of spermatids in P32 spermatids. The following pseudocoloring is used: spermatid nuclei, pink; acrosomes, blue; Golgi, green; transport vesicles, red; endoplasmic reticula, yellow. Acr, acrosome; PAG, proacrosomal granule; ST, spermatid. Scale bars: 500 nm. (D) Quantification of acrosome morphology from EM micrographs. (E) Immunostaining against SP-10 in testis sections in P23 mice. The most advanced spermatids in S8/*Fis1* testes are shown. The steps of spermatid development are indicated. Scale bars: 20 µm. (F) Schematic of the spermatogenic arrest in S8/*Fis1* mice. *Fis1* GCs form predominantly at steps 2-4 of spermatid development, but can also form at steps 5-8. Note that in *Fis1* mutants, no step 9 elongating spermatids are found in individual spermatids or GCs. Data are represented as mean±s.e.m. ****P≤0.0001; ***P≤0.001. For statistical tests used, see the Materials and Methods section.

The analyses described above indicate that S8/*Fis1* mice complete meiosis, but exhibit spermatogenic arrest during round spermatid development. To determine the exact stage of the spermatid arrest in S8/*Fis1* mice, we first performed PAS staining in juvenile P28 mice, which are undergoing the first round of spermatogenesis. The PAS reaction clearly marks the acrosome in control spermatids (Fig. 4A) (Osuru et al., 2014). In *Fis1*-null mutants, round spermatids were present, but the PAS-positive structures appeared fragmented and widely dispersed, lacking the compact crescent shape found in control cells. To verify that the aberrant PAS structures in the mutant tubules were related to the acrosome, we stained testis sections with the SP-10 acrosome marker described above. Indeed, the SP-10-positive acrosome structures in mutant GCs were fragmented and dispersed throughout the cytoplasm (Fig. 4B).

To visualize acrosome ultrastructure, we performed electron tomography in testis sections (Fig. 4C,D). Trans Golgi cisternae in control spermatids were found juxtaposed against the acrosome (Fig. 4C, Movie 1), consistent with previous observations that the acrosome is largely a Golgi-derived organelle (Berruti and Paiardi, 2011). In contrast, S8/*Fis1* GCs lacked fully formed acrosomes and contained disorganized Golgi cisternae and proacrosomal granules (Fig. 4C, Movie 2), indicating a defect in acrosome biogenesis, morphogenesis or maintenance. Given the immature stage of the acrosomes (steps 2-4) found in giant cells, this analysis suggests that spermatids form giant cells early in their development.

To define more precisely the stage of the spermatogenic arrest in S8/*Fis1* mice, we examined younger (P23) mice in which spermatids were first forming (Fig. 4E). We determined the spermatid steps using the acrosome marker SP-10. In P23 S8/*Fis1* mice, individual spermatids at step 2-4 could be readily found. More advanced individual spermatids at steps 5-8 could also occasionally be seen. These observations indicated that spermatids and their acrosomes can only progress to early stages in the absence of *Fis1*. Strikingly, no step 9 elongating spermatids could be found in juvenile or adult S8/*Fis1* testes. Taken together, these data suggest that S8/*Fis1* spermatids coalesce into GCs predominantly at steps 2-4, with a smaller population perhaps forming GCs at steps 5-8 (Fig. 4F).

### Mitochondrial defects in *Fis1*-null round spermatids and giant cells

**Fig. 5.**
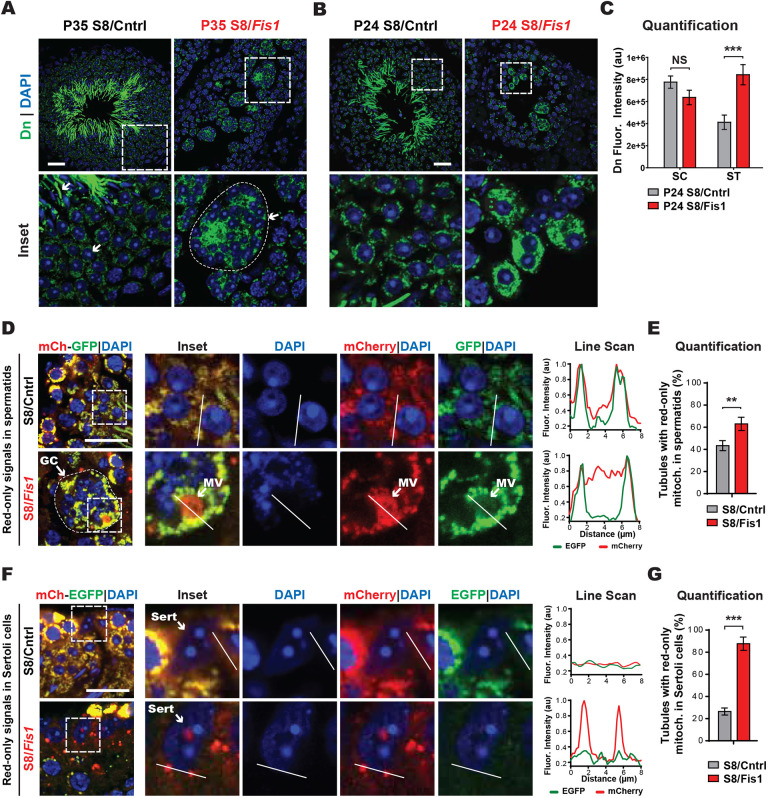
***Fis1*-null giant cells exhibit mitochondrial accumulation and perturbed mitophagy.** (A) Mitochondrial content of germ cells at P35. Dendra2 (Dn)-positive mitochondria were visualized in testes sections counterstained with DAPI. The rod-like structures in the center of control seminiferous tubules are mitochondria in the midpiece of elongating spermatids, which are absent in the mutant. Note the accumulation of Dendra2-positive mitochondria in S8/*Fis1* GCs. Dashed line encircles a spermatid giant cell. Scale bar: 20 µm. (B) Mitochondrial content of germ cells at P24. Dendra2-positive mitochondria were visualized in testes sections counterstained with DAPI. Note the accumulation of Dendra2-positive mitochondria in S8/*Fis1* spermatids. Scale bar: 20 µm. (C) Quantification of mito-Dendra2 fluorescence intensity. Dendra2 intensity was normalized to the number of nuclei. (D) Analysis of mitophagy in testis sections from S8-mCherry-EGFP mice, counterstained with DAPI. Cytosolic mitochondria have both red and green fluorescence, whereas mitochondria in acidic compartments are red only. Line scans of the indicated regions are shown to the right. MV, mitophagic vesicle. Scale bar: 20 µm. (E) Quantification of mitophagy in spermatids. (F) Analysis of heterophagy in testis sections from S8-mCherry-EGFP mice, counterstained with DAPI. Note that Sertoli cells (Sert) contain red-only mitochondria, which are derived from germ cells. Line scans of the indicated regions are shown to the right. Scale bar: 20 µm. (G) Quantification of red-only signals in Sertoli cells. All data are from P35 mice. Data are represented as mean±s.e.m. ***P≤0.001; **P≤0.01. For statistical tests used, see the Materials and Methods section. au, arbitrary unit; NS, not significant.

To understand the cellular mechanism for these spermatogenic defects, we first examined mitochondria using a mouse line expressing mitochondrially localized Dendra2 (mito-Dendra2) (Pham et al., 2012). At P35, we noticed a marked increase in mito-Dendra2 fluorescence intensity in S8/*Fis1* GCs compared with control spermatids (Fig. 5A), indicating mitochondrial accumulation. To rule out the possibility that the mitochondrial accumulation is a consequence of GC formation, we analyzed mito-Dendra2 fluorescence in P24 S8/*Fis1* mice, in which individual round spermatids and GCs with only a few nuclei could be readily identified (Fig. 5B). To account for binucleated and multinucleated spermatids in P24 S8/*Fis1* mice, we normalized the mito-Dendra2 fluorescence intensity to the number of spermatid nuclei. This quantification revealed that S8/*Fis1* spermatids have a two-fold increase in mito-Dendra2 fluorescence intensity (Fig. 5C). S8/*Fis1* spermatocytes did not have an increase in mito-Dendra2 signal, indicating that the increased mitochondrial signal is specific to spermatids.

Because *Fis1* was previously implicated in mitophagy, we tested whether the increased mito-Dendra2 fluorescence in S8/*Fis1* GCs could be due to defective mitophagy. To this end, we generated conditional mitophagy reporter mice, which express a mitochondrially targeted mCherry-EGFP fusion protein, preceded by a floxed stop cassette (Fig. S4A,B). In acidic compartments mitochondrial EGFP fluorescence is selectively quenched, whereas mCherry fluorescence remains intact. Thus, mitochondria that are undergoing mitophagy exhibit red-only fluorescence. After crossing the conditional mitophagy reporter mice to the *Stra8-Cre* driver, we found an increase in the number and size of red-only signals in S8/*Fis1* GCs (Fig. 5D,E). Combined with the mitochondrial accumulation in *Fis1* mutants, these data suggest that there is a disruption of mitophagy.

To determine whether the accumulated mitochondria in GCs are dysfunctional, we measured the activity of the respiratory chain complexes using COX/SDH enzyme histochemistry (Ross, 2011) in testis sections. We found that S8/*Fis1* GCs exhibited increased staining for both COX and SDH, indicating increased respiratory chain complex IV and II activity (Fig. S4C,D). The increased respiratory chain activity can reflect increased activity at the individual organelle level or may simply reflect the increased mitochondrial content in GCs. Nevertheless, the observation that S8/*Fis1* GC mitochondria are not dysfunctional suggests that *Fis1*-induced mitophagy acts to remove excess, not dysfunctional mitochondria. To further verify that *Fis1*-deficient mitochondria are functional, we measured mitochondrial membrane potential by MitoTracker Red staining (Fig. S4E,F). For unknown reasons, wild-type haploid spermatids did not stain with MitoTracker Red. In contrast, earlier germ cell types had robust MitoTracker Red staining that colocalized with mito-Dendra2. In these younger germ cell types, *S8/Fis1* mutants did not have any obvious reduction in MitoTracker Red staining, indicating intact membrane potential (Fig. S4F). Because *Fis1* has also been shown to regulate peroxisome morphology (Kobayashi et al., 2007; Koch et al., 2005), we examined whether *Fis1* GCs also contained aberrant peroxisomes (Fig. S4G). Control spermatids and *Fis1* giant cells had little or no staining with the PEX14 peroxisome marker. We observed a slight increase in PEX14 staining in spermatocytes and a more robust increase at the tubule periphery around spermatogonia and Sertoli cells.

Interestingly, red-only mitophagy signals were sometimes seen outside of germ cells, within the Sertoli cell cytoplasm (Fig. 5F). Because the mitophagy reporter is driven by *Stra8-Cre* expression in germ cells, the red-only signals in Sertoli cells should be derived from germ cell mitochondria. Red-only mitophagy signals within the Sertoli cell cytoplasm could reflect hitherto uncharacterized transcellular degradation of germ cell mitochondria by Sertoli cells (transmitophagy), as occurs between glial cells and neurons (Davis et al., 2014). Alternatively, it may reflect phagocytic degradation of apoptotic germ cells (heterophagy), which is known to occur in Sertoli cells. In addition to the increased mitophagy in spermatids, *Fis1* mutant tubules also exhibit increased mitophagy signals in the Sertoli cell cytoplasm (Fig. 5G).

### S8/*Fis1* spermatid giant cells exhibit aberrant accumulation of autophagic structures

**Fig. 6.**
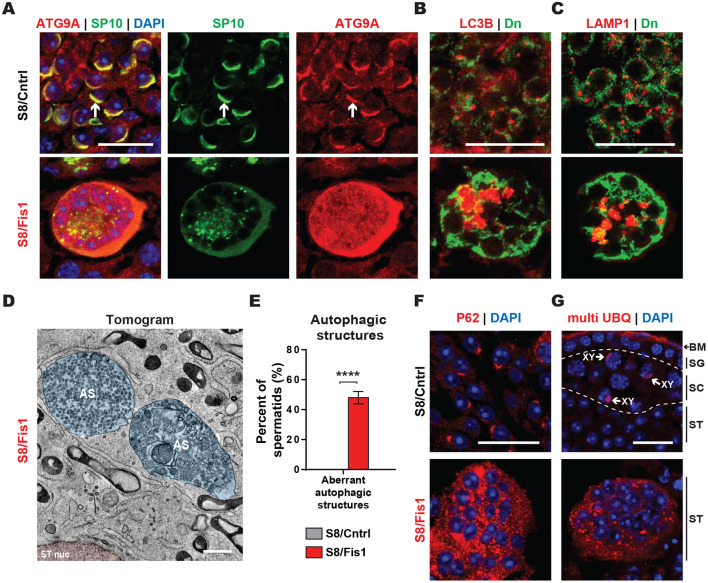
**S8/*Fis1* spermatid giant cells exhibit aberrant accumulation of autophagic structures.** (A) Immunostaining of testis sections with the early autophagic marker ATG9A. Note the localization of ATG9A to the acrosome (white arrows) and the accumulation of ATG9A in mutant spermatids. Scale bar: 20 µm. (B) Immunostaining of testis sections with the autophagy marker LC3B. Scale bar: 20 µm. (C) Immunostaining of testis sections with the lysosome marker LAMP1. Scale bar: 20 µm. (D) Electron tomogram of a P36 *Fis1* GC. The following pseudocolors are used: Nucleus, pink; autophagic structures, blue. AS, autophagic structures; ST nuc, spermatid nucleus. Scale bar: 500 nm. (E) Quantification of aberrant autophagic structures from EM micrographs. (F) Immunostaining of testis sections with an antibody against the autophagy marker P62. Note the accumulation and aggregation of P62 in mutant spermatids. Scale bar: 20 µm. (G) Immunostaining of testis sections with an antibody against monoubiquitin and multiubiquitin chains (K29-, K48- and K63-linked). BM, basement membrane; SC, spermatocyte; SG, spermatogonia; ST, spermatid. As expected, control spermatocytes have ubiquitin staining in the XY body, and control spermatids lack ubiquitin signal. Scale bar: 20 µm. All data are from P35 animals, unless otherwise indicated. Data are represented as mean±s.e.m. ****P≤0.0001. For statistical tests used, see the Materials and Methods section.

Our analysis with mitophagy reporter mice demonstrated that *Fis1*-null GCs accumulate large mitophagic vesicles (Fig. 5D,E). We therefore checked whether *Fis1*-null GCs exhibit a block in autophagic flux. We first examined the expression of the early autophagy marker ATG9A, which is an integral membrane protein on vesicles that generate autophagosomes (Imai et al., 2016) (Fig. 6A). Interestingly, in control mice, ATG9A localizes to the acrosome. In contrast, ATG9A in S8/*Fis1* GCs accumulates throughout the cytoplasm. We next looked at the expression patterns of LC3B (MAP1LC3B) and LAMP1, which mark autophagosomes and lysosomes, respectively (Fig. 6B,C). Compared with control, *Fis1* mutants exhibited an accumulation of large LC3B and LAMP1 aggregates, consistent with a block in autophagic flux. To visualize the ultrastructure of these autophagic structures, we performed electron tomography in testis sections. Consistent with the immunostaining data, our electron microscopy (EM) analysis identified massive autophagic structures (Fig. 6D,E, Fig. S5A,B, Movie 3). These autophagic structures are circumscribed by a single membrane and therefore are most likely to be autolysosomes.

We next checked the expression pattern of a widely used marker of autophagic flux, P62 (SQSTM1) (Katsuragi et al., 2015) (Fig. 6F). We found that P62 is massively accumulated in S8/*Fis1* GCs. Because P62 is an autophagy cargo adaptor that is degraded along with cargo, high accumulation of P62 suggests a block in autophagic turnover. Because autophagy utilizes ubiquitin tags, we also probed testis sections with an antibody against multi-ubiquitin (Fig. 6G). Consistent with previous reports (Baarends et al., 2005; Lu et al., 2010), we found ubiquitin signal on the XY body in control spermatocytes (Fig. 6G, arrows), indicating specificity of the antibody. Control round spermatids lacked any ubiquitin signal, whereas S8/*Fis1* GCs displayed a pronounced accumulation of ubiquitin aggregates. Taken together, these data indicate that *Fis1* is required for autophagic degradation of spermatid mitochondria.

### *Fis1*-null giant cells accumulate aberrant mitochondria

**Fig. 7.**
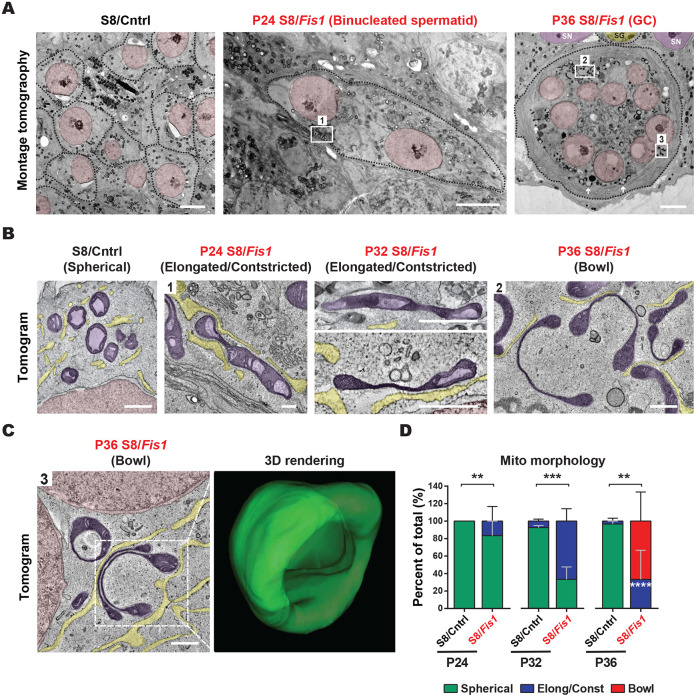
***Fis1*-null giant cells accumulate aberrant mitochondria.** (A) EM montage tomography in testis sections showing individual round spermatids in the control sample, a binucleated spermatid in the P24 mutant, and a multinucleated giant cell in the P36 mutant. White arrows indicate a few examples of aberrant mitochondria. The following pseudocolors are used: spermatid nuclei, pink; Sertoli cell nuclei, purple; spermatogonium nucleus, yellow. SG, spermatogonium; SN, Sertoli nucleus. Scale bars: 5 µm. (B) EM tomograms from the regions indicated in Fig. 7A. Note the highly aberrant mitochondria in the mutant. Scale bars: 500 nm. See also Movies 3-6. (C) EM tomogram of a P36 *Fis1* GC from region 3 indicated in Fig. 7A. The panel on the right shows a 3D rendering generated from serial sections. Scale bar: 500 nm. See also Movie 7. (D) Quantification of mitochondrial morphology from EM micrographs. Data are represented as mean±s.e.m. ***P≤0.001; **P≤0.01. For statistical tests used, see the Materials and Methods section.

To examine the ultrastructure and morphology of the accumulated mitochondria in *Fis1*-null spermatids, we performed electron tomography in testis sections (Fig. 7). To visualize mitochondria within entire GCs, we first performed montage tomography (Fig. 7A). The vast majority of control round spermatids had small and spherical mitochondria, as described previously (Varuzhanyan et al., 2021). In contrast, mitochondria in S8/*Fis1* GCs had highly aberrant ultrastructure. Even in early, binucleated spermatids, aberrant mitochondrial constrictions could be found. By P36, S8/*Fis1* giant cells were filled with peculiar dumbbell-shaped mitochondria (Fig. 7A). To understand how these unusually shaped mitochondria formed in S8/*Fis1* GCs, we generated high-resolution tomograms from P24, P32 and P36 spermatids (Fig. 7B-D, Movies 4-7) to track mitochondrial morphology through time. In control round spermatids, mitochondria were almost always small and spherical, regardless of the age of the animal and the developmental step of the spermatid (Fig. 7B, Movie 4). In contrast, in P24 S8/*Fis1* sections, elongated mitochondria with unusual constrictions could be seen in ∼20% of cells (Fig. 7B,D, Movie 5). By P32, the number of mutant spermatids with elongated/constricted mitochondria increased to 70% (Fig. 7B,D, Movie 6). By P36, mutant mitochondria displayed severe and lengthy constrictions near the center, and a bulbous region at each end to yield a dumbbell shape (Fig. 7B,D, Movie 7). To visualize morphology of the unusual dumbbell-shaped organelles better, we performed serial-section electron tomography in P36 *Fis1* GCs and generated 3D renderings of entire mitochondria (Fig. 7C, Movie 8). This 3D analysis revealed that mutant mitochondria in P36 giant cells form bowl-like structures with a thickened rim. Taken together, these data suggest that mutant mitochondria elongate and constrict before transforming into bowl-like structures.

## DISCUSSION

Given the importance of mitochondrial function during spermatogenesis, we have used the male germ cell system to study developmentally regulated mitochondrial dynamics. We previously reported that mitofusin*-*mediated mitochondrial fusion is required for differentiation of spermatogonia and progression of spermatocytes through meiosis (Varuzhanyan et al., 2019). In addition, we showed that MFF-mediated fission generates small mitochondrial fragments to enable the organization of the mitochondrial sheath within the spermatozoon midpiece (Varuzhanyan et al., 2021). Although *Mff* mutant mice had pronounced mitochondrial constrictions in round spermatids, they did not exhibit spermatid arrest or GC formation. Although regulating the balance between fusion and fission is an effective mechanism for changing mitochondrial shape, it does not change total mitochondrial mass unless coupled to biogenesis or turnover. This study shows that mitochondrial turnover, via *Fis1*-dependent mitophagy, plays an important role in spermatid maturation (Fig. S6).

Spermatid multinucleation occurs in multiple species and in response to various stresses (Brauns et al., 2019; Elliott et al., 2010; Fawcett et al., 1959; Holstein and Eckmann, 1986; Kanwar et al., 1971; MacGregor et al., 1990; Morton et al., 2016; Neumann and Schenck, 1977; Orazizadeh et al., 2010; Rao and Srivastava, 1967; Rotter et al., 1993; Singh and Abe, 1987a; Yamada et al., 2006). Two observations argue that the mitochondrial defects in *Fis1* mutant mice are likely the cause and not the consequence of giant cell formation. First, mitochondrial constrictions and accumulation are present in individual and binucleated spermatids, prior to giant cell formation (Fig. 7A,B). Second, such mitochondrial abnormalities are not found in giant cells caused by other perturbations (Brauns et al., 2019; Holstein and Eckmann, 1986; Morton et al., 2016; Singh and Abe, 1987; Yamada et al., 2006).

Our results raise several important issues that should be addressed in future studies. First, our work shows that mitophagy controls the density of mitochondria in spermatids, but it is unclear whether the mitochondria being removed are functionally defective. The accumulated mitochondria of S8/*Fis1* spermatids show severe morphological abnormalities, suggestive of physiological defects. Nevertheless, histochemical staining shows that electron transport chain activities are intact, although these crude measures can miss subtle defects. It would be of interest to determine whether mitophagy in this system is used primarily to control mitochondrial density in the cell, versus also playing a role in mitochondrial quality control. Second, it remains to be determined why *Fis1* knockout spermatids collapse into multinucleated GCs after completing the early stages of maturation. During normal male germ cell division, cytokinesis does not proceed to completion. The midbodies persist and transform into stable intracellular bridge structures that connect germ cells together to form a syncytium (Greenbaum et al., 2011). It has been speculated that GC formation can result from disruption of intracellular bridges, and some evidence implicates defective cytokinesis (Brill et al., 2000; Giansanti et al., 2004; Greenbaum et al., 2011). It will be of interest to determine whether *Fis1* has a connection to these events, or whether GC formation is a generic response to certain forms of spermatid dysfunction. Finally, it will be interesting to determine the cause of the robust, ectopic expression of γH2AX in *Fis1*-null spermatids. Phosphorylation of the histone H2AX at serine 139 to form γH2AX is a well-established response to DNA damage (Mah et al., 2010; Rogakou et al., 1998). Therefore, future studies should explore whether *Fis1*-null spermatids exhibit DNA damage, and if so, the trigger for this state.

## MATERIALS AND METHODS

### Generation of mice

All mouse experiments were approved by the California Institute of Technology (Caltech) Institutional Animal Care and Use Committee. The conditional *Fis1* mouse (*Fis1^loxP^*) was generated at the Janelia research campus. With *loxP* sites flanking exon 2, Cre-mediated recombination results in a frameshift mutation that produces a null allele. S8/Control and S8/*Fis1* mice were generated by crossing *Stra8-Cre*; *Fis1^+/Δ^* mice to *Fis1^loxP^*^/*loxP*^; *Rosa26^PhAM(loxP/loxP)^* mice. Wild-type and heterozygous littermates were used as controls. The *Stra8-Cre* driver (Jackson Laboratory #017490) (Sadate-Ngatchou et al., 2008) and the mito-Dendra2 allele (Pham et al., 2012) have both been described previously. All mice were maintained on a C57B6 genetic background. In the mitophagy reporter mouse [*Rosa26*^mCherry-EGFP(*loxP*/*loxP*)^], a tandem repeat of the mitochondrial targeting sequence of COX IV was attached to the N terminus of mCherry-EGFP. The mito-mCherry-EGFP expression cassette has the CAG promoter and a floxed polyadenylation signal-Neo-STOP cassette, and was inserted into intron 1 of the ROSA26 locus. To generate germ cell-specific mitophagy reporter mice, we crossed *Stra8-Cre*; *Fis1*^+/Δ^ mice to *Fis1^loxP^*^/*loxP*^; *Rosa26*^mCherry-EGFP(*loxP*/*loxP*)^ mice.

### Epididymal sperm counting

Mice were euthanized at P56, and epididymides were dissected and thoroughly minced in 1.7 ml microcentrifuge tubes containing 1 ml PBS. Samples were incubated at 37°C for 20 min to allow sperm to swim out and 900 µl of the supernatant was transferred into a fresh microcentrifuge tube. For sperm counting and morphology analysis, samples were allowed to settle for several hours to allow sperm to stop swimming before counting on a hemocytometer. Sperm counts were normalized to the weight of the epididymides of each mouse.

### PAS staining

After dissection, testes were fixed in Bouin's fixative overnight at 4°C, dehydrated in a 30-90% ethanol gradient, cleared in xylene, and embedded in paraffin. Tissue blocks were sectioned at 7 µm, deparaffinized and rehydrated before staining. Briefly, slides were incubated with 1% periodic acid [Electron Microscopy Sciences (EMS), 19324-10] for 30 min at room temperature (RT), washed in running water for 5 min, then rinsed in deionized water. Slides were incubated with Schiff's reagent (EMS, 260582-05) for 30 min at RT and washed as described above before counterstaining with Hematoxylin Gill 2 for 30 s at RT. Slides were washed in running water for 1 min, dehydrated with ethanol, cleared with xylene, then mounted using Cytoseal XYL mounting media (Thermo Fisher Scientific, 22-050-262).

### Dissociation of testicular cells for MitoTracker Red staining

Testes were dissociated from juvenile males (1-2 months old) as described previously (Gaysinskaya and Bortvin, 2015; Gaysinskaya et al., 2014). The cell suspension was passed through a 100 µm nylon cell strainer, pelleted at 150 ***g*** for 5 min, and plated onto 12 mm coverslips pre-coated with Cell-Tak (Corning, 354240). Coverslips were placed in a 12-well plate and centrifuged at 1000 rpm (230 ***g***) for 5 min to promote adhesion. Cells were stained with MitoTracker Red CMXRos (Thermo Fisher Scientific, M7512) (150 nM) at 35°C for 30 min, washed in PBS, then fixed in 3.7% formalin solution for 10 min at 37°C. After washing, cells were permeabilized in acetone at −20°C for 10 min. Cells were washed and immediately immunostained with SP-10 to identify round spermatids.

### Immunofluorescence

For immunostaining of tissue sections, testes were cut at the poles, fixed in 4% paraformaldehyde for 4 h at 4°C, incubated with 30% sucrose in PBS overnight at 4°C (or until tissues sank), incubated in a 1:1 solution of 30% sucrose in PBS and optimal cutting temperature (OCT) embedding medium (Thermo Fisher Scientific, NC9636948) for 15-30 min, then embedded in OCT medium and frozen in dry ice. Tissue blocks were sectioned at 10 µm onto glass slides, dried overnight, and stored at −80°C until ready for immunostaining. Frozen slides were briefly thawed at room temperature, rehydrated in PBS, permeabilized with 0.15% Triton X-100 for 15 min, and blocked for 1 h using Blocking Buffer (10% fetal bovine serum, 3% bovine serum albumin, 0.05% Triton X-100 in PBS). Slides were incubated with primary antibodies in a humidified chamber overnight at 4°C, washed three times in PBS for 15 min each, then incubated with secondary antibodies in a humidified chamber for 2.5 h at RT. Slides were counterstained with DAPI, washed as described above, mounted with Fluoro-Gel (EMS; 17985-10), coverslipped, sealed with nail polish, and stored at 4°C before imaging.

### Apoptotic cell labeling

To label apoptotic nuclei, the TUNEL assay was performed in paraformaldehyde-fixed, OCT-embedded testis sections using the ApopTag Red In Situ Apoptosis Detection Kit (Millipore, S7165) according to the manufacturer's protocol. Nuclei were counterstained with DAPI.

### Electron microscopy and dual-axis tomography

Mouse testes were dissected and immediately fixed with cold 3% glutaraldehyde, 1% paraformaldehyde, 5% sucrose in 0.1 M sodium cacodylate trihydrate. Pre-fixed pieces of tissue were rinsed with fresh cacodylate buffer and placed into brass planchettes (Type A; Ted Pella) prefilled with 10% Ficoll in cacodylate buffer. Samples were covered with the flat side of a Type B brass planchette and rapidly frozen with an HPM-010 high-pressure freezing machine (Leica Microsystems). The frozen samples were transferred under liquid nitrogen to cryotubes (Nunc) containing a frozen solution of 2.5% osmium tetroxide, 0.05% uranyl acetate in acetone. Tubes were loaded into an AFS-2 freeze-substitution machine (Leica Microsystems) and processed at −90°C for 72 h, warmed over 12 h to −20°C, held at that temperature for 8 h, then warmed to 4°C for 2 h. The fixative was removed, and the samples were rinsed four times with cold acetone, and then were infiltrated with Epon-Araldite resin (EMS) over 48 h. Samples were flat-embedded between two Teflon-coated glass microscope slides, and the resin polymerized at 60°C for 24-48 h.

Flat-embedded testis samples were observed with a stereodissecting microscope, and appropriate regions were extracted with a microsurgical scalpel and glued to the tips of plastic sectioning stubs. Semi-thick (400 nm) serial sections were cut with a UC6 ultramicrotome (Leica Microsystems) using a diamond knife (Diatome). Sections were placed on Formvar-coated copper-rhodium slot grids (EMS) and stained with 3% uranyl acetate and lead citrate. Gold beads (10 nm) were placed on both surfaces of the grid to serve as fiducial markers for subsequent image alignment. Grids were placed in a dual-axis tomography holder (Model 2040, E.A. Fischione Instruments) and imaged with a Tecnai TF30ST-FEG transmission electron microscope (300 KeV) equipped with a 2k×2k CCD camera (XP1000; Gatan). Tomographic tilt-series and large-area montaged overviews were acquired automatically using the SerialEM software package (Mastronarde, 2005). For tomography, samples were tilted ±64° and images collected at 1° intervals. The grid was then rotated 90° and a similar series taken about the orthogonal axis. Tomographic data was calculated, analyzed and modeled using the IMOD software package (Kremer et al., 1996; Mastronarde, 2008) on Apple MacPro computers.

### Confocal, bright-field imaging, and image processing

Confocal fluorescence images were acquired using an inverted Zeiss LSM 710 confocal microscope with a 60× Plan-Apochromat objective. Bright-field images were acquired using an upright Nikon Eclipse Ni-E fluorescence microscope equipped with a Ds-Ri2 camera and CFI Plan Apochromat Lambda objectives. For PAS histology images, *z*-stacks were acquired, and ‘all-in-focus’ images were created using the NIS Elements Extended Depth of Focus plugin. All images were processed using ImageJ. All image modifications were performed on entire images (no masking was used) and were performed identically between genotypes.

### Replicates and statistical reporting

Pairwise comparisons were made using the Student's *t*-test. When multiple pairwise comparisons were made from the same dataset, *P*-values were adjusted using the Bonferroni correction. For comparisons of more than two means, one-way ANOVA was used, followed by Tukey's post-hoc test. Number of mice and replicates are indicated in figure legends. All outliers were included in the analysis. All data are represented as mean±s.e.m. and statistical significance indicated as follows: ****P≤0.0001; ***P≤0.001; **P≤0.01; *P≤0.05.

### Quantification from testis sections

Seminiferous tubules were scored from 10-µm-thick testis sections using the subcellular markers described in the main text. Quantification was restricted to germ cells within round transverse sections of seminiferous tubules. For each genotype, at least 50 transverse sectioned seminiferous tubules were quantified from at least three mice.

### Antibodies

We used the following antibodies for immunofluorescence: rabbit anti-γH2AX (ab11174, Abcam, 1:2000); mouse anti-γH2AX (ab26350, Abcam, 1:500); guinea pig anti-SP-10 (gift from Prabhakara P. Reddi, University of Virginia, Charlottesville, VA, USA; 1:1000); rabbit anti-LC3B (2775S, Cell Signaling Technology, 1:200); rabbit anti-LAMP1 (ab24170, Abcam, 1:200); rabbit anti-RAB7A (ab137029, EPR7589, Abcam, 1:200); rabbit anti-Na/K-ATPase (ab76020, Abcam, 1:200); rabbit anti-ATG9A [ab108338, EPR2450(2) Abcam, 1:500]; mouse anti-multi-ubiquitin (D058-3, MBL, 1:500); rabbit anti-FIS1 (10956-1-AP, Proteintech, 1:50); rabbit anti-P62 (PM045, MBL, 1:300). Secondary antibodies used were: donkey anti-rabbit 488 (Invitrogen, A-21206, 1:400); donkey anti-rabbit 546 (Invitrogen, A10040, 1:400); donkey anti-rabbit 555 (Invitrogen, A32794, 1:800); donkey anti-mouse 546 (Invitrogen, A10036, 1:400); donkey anti-mouse 488 (Invitrogen, A21202, 1:400); goat anti-guinea pig FITC (Invitrogen A18776, 1:400); goat anti-guinea pig HRP (Invitrogen, A18769, 1:400).

## Supplementary Material

Supplementary information

Reviewer comments
