# Supplementary material for: Fis1 ablation in the male germline disrupts mitochondrial morphology and mitophagy, and arrests spermatid maturation
Source: Development. 2021 Aug 12;148(16):dev199686. doi: 10.1242/dev.199686 (PMC8380467; doi:10.1242/dev.199686)
Supplement: Supplementary information [file develop-148-199686-s1.pdf]

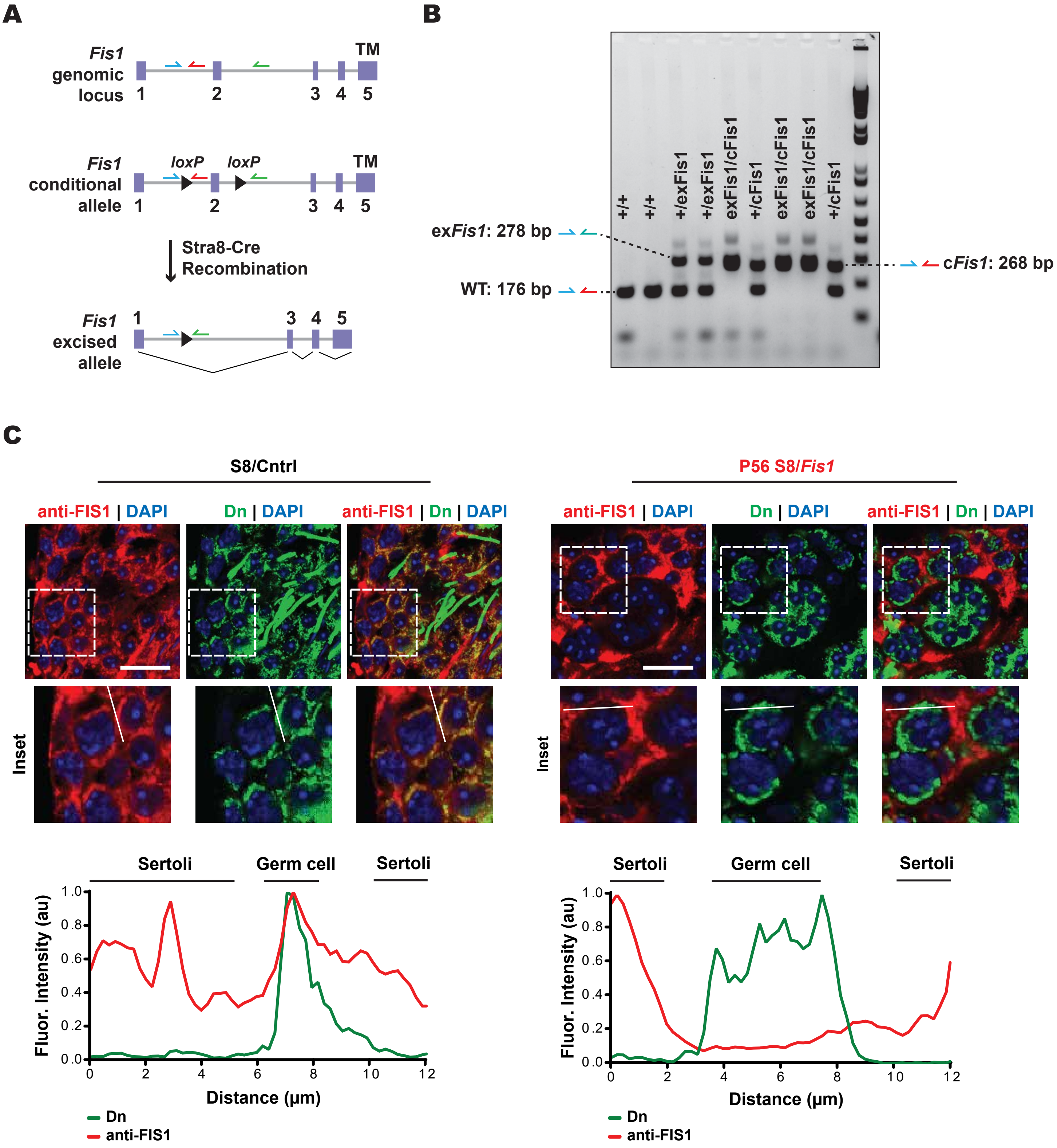

Development • Supplementary information

Fig. S1. Related to Fig. 1

(A) Schematic of the conditional (floxed) and excised *Fis1* alleles. In the conditional *Fis1* allele, *loxP* sites flank exon 2. After *Cre*-mediated recombination, deletion of exon 2 causes a frameshift in the *Fis1* open reading frame. Genotyping oligos are indicated.

(B) Genotyping from mouse tails. Genotyping oligos and the predicted fragment sizes are indicated. See also Fig. S1A.

(C) Immunostaining of testis sections with an antibody against FIS1. Note that Dendra2-positive germ cell mitochondria in the *Fis1* mutants (right panel) are negative for FIS1 immunostaining. Line scans of the indicated regions are shown at the bottom. The regions corresponding to germ and Sertoli cell mitochondria are indicated. Scale bars, 20 µm.

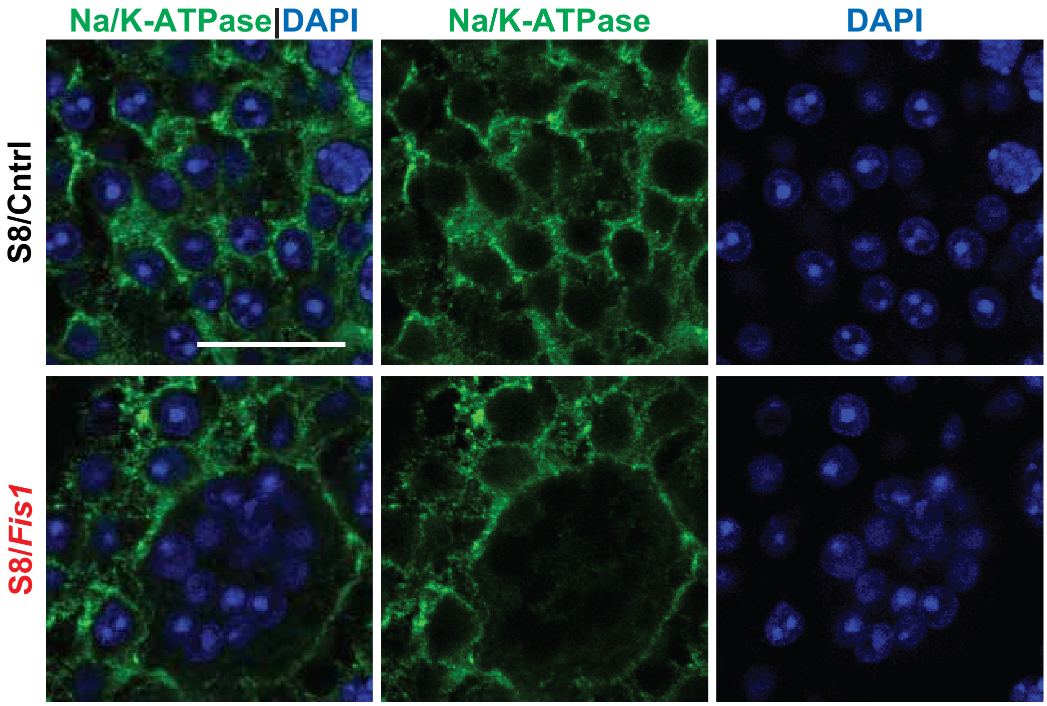

Fig. S2. Related to Fig. 2

Immunostaining germ cell boundaries using an antibody against the plasma membrane marker Na/K-ATPase. The bottom panel (mutant) shows that the nuclei within GCs are not separated by plasma membrane. Scale bar, 20 µm.

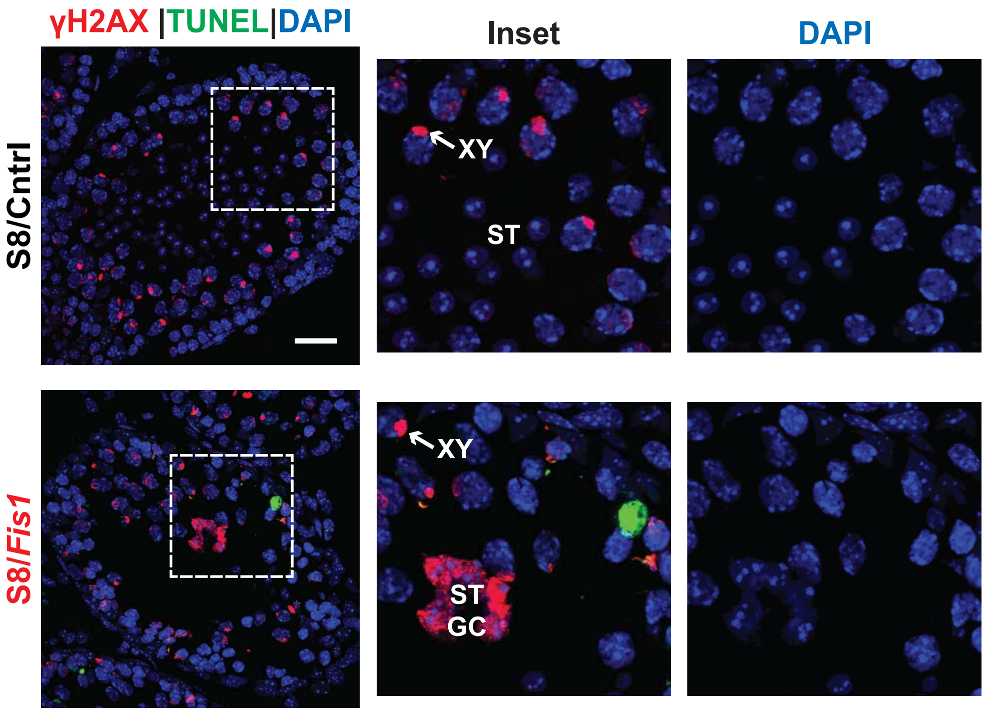

Fig. S3. Related to Fig. 3

Analysis of apoptosis in *Fis1* GCs. TUNEL assay was performed in P35 testis sections co-stained with an antibody against γH2AX and DAPI. Note that the aberrant *Fis1* GC is not TUNEL-positive. ST, spermatid; GC, giant cell. Scale bar, 20 µm.

Development • Supplementary information

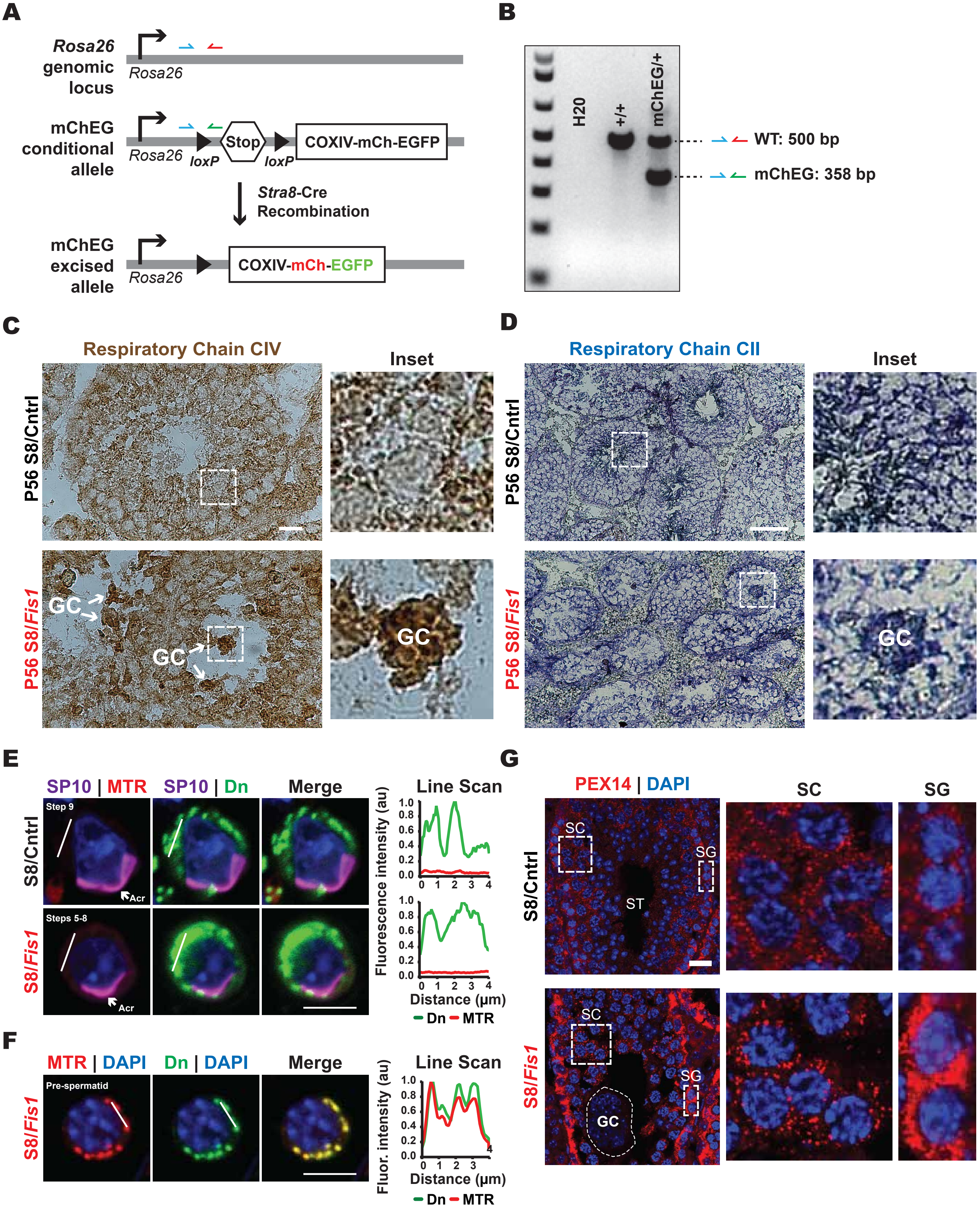

Development • Supplementary information

Fig. S4. Related to Fig. 5

(A) Schematic showing the generation of germ cell-specific mitophagy reporter mice. Genotyping oligos are indicated.

(B) Genotyping from mouse tails. Genotyping oligos and predicted band sizes are indicated. See also Fig. S5A.

(C) COX (Complex IV) enzyme histochemistry in P56 testis sections.

(D) SDH (Complex II) enzyme histochemistry in P56 testis sections.

(E) MitoTracker Red Staining in dissociated spermatids. SP10 marks the acrosome, which is unique to spermatids. Scale bar, 5 µm

(F) MitoTracker Red Staining in pre-spermatid S8/*Fis1* germ cells. Scale bar, 5 µm

(G) Immunostaining of testis sections with the PEX14 peroxisome marker. SC, spermatocyte; SG, spermatogonia. Scale bar, 20 µm.

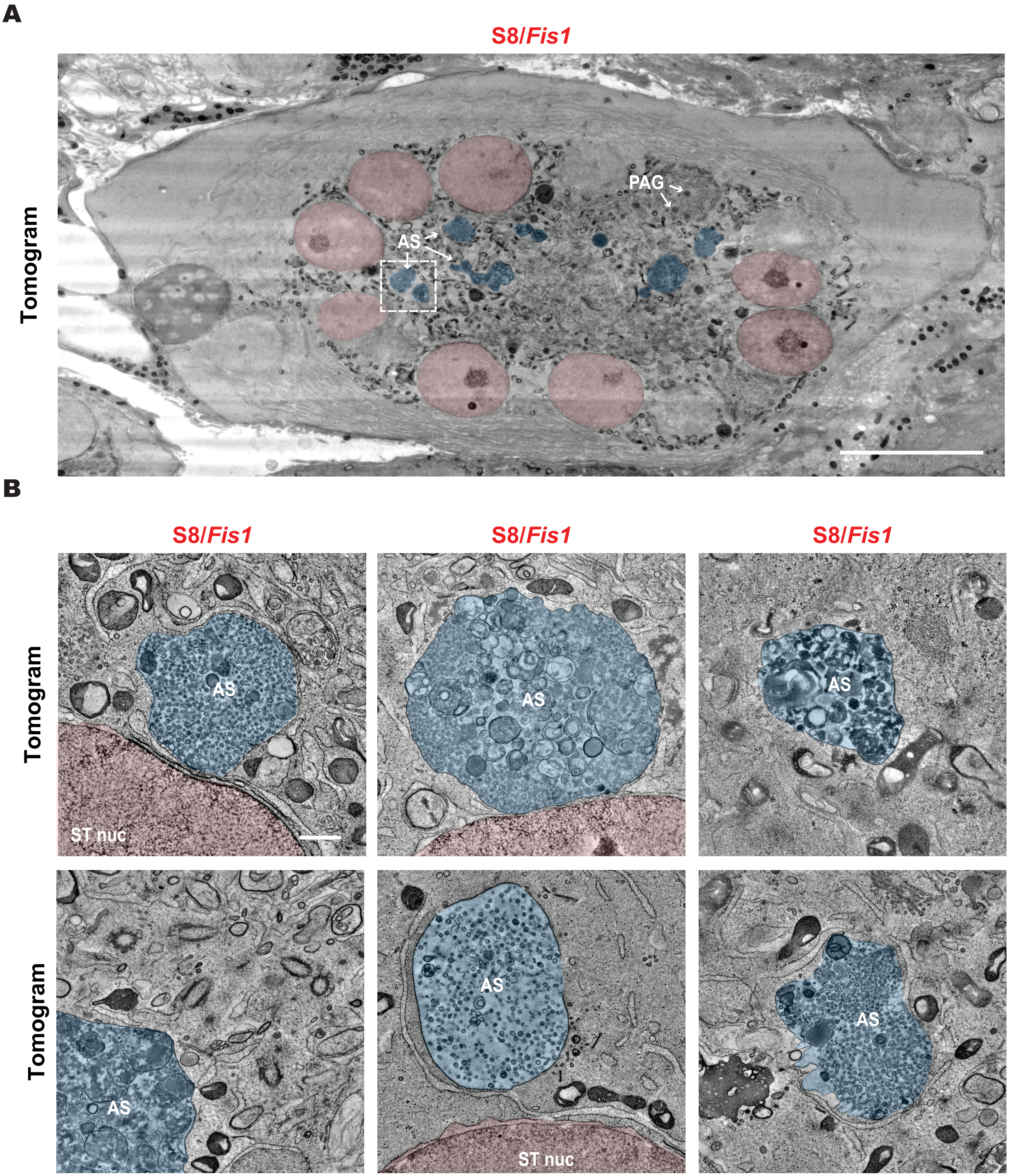
A
S8/Fis1
Tomogram
PAG
AS
B
S8/Fis1
S8/Fis1
S8/Fis1
Tomogram
AS
ST nuc
AS
AS
Tomogram
AS
AS
ST nuc
AS

Fig. S5. Related to Fig. 6.

(A) Montage tomography from an S8/*Fis1* GC. AS, autophagic structures; PAG, proacrosomal granules. Scale bar 10 µm.

(B) EM tomograms showing autophagic structures in *Fis1* giant cells (GCs). The following pseudocolors are used. Nuclei, pink; autophagic structures, blue. Scale bar, 500 nm.

Development • Supplementary information

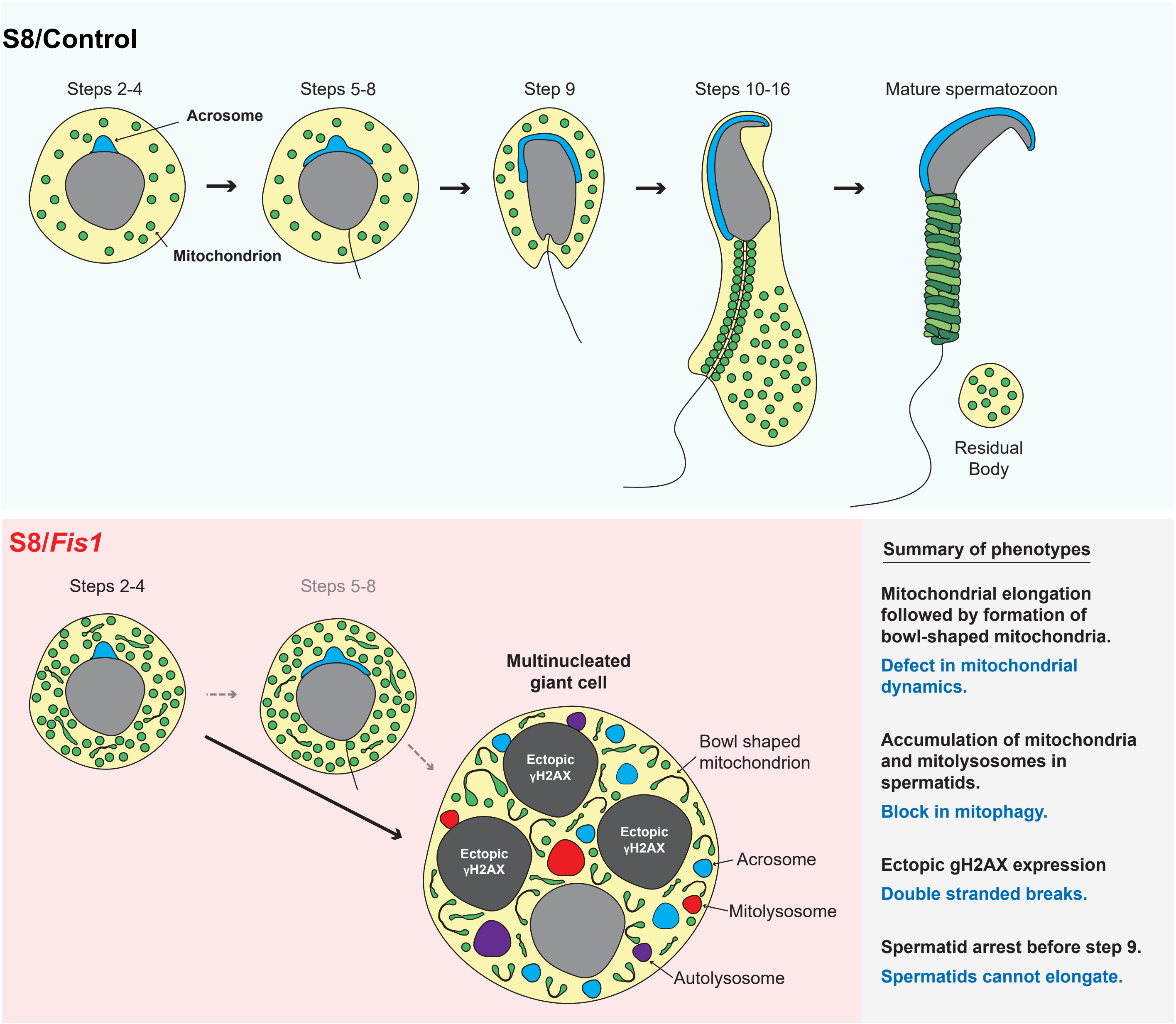

Fig. S6. Related to Fig. 7.

Schematic of the spermatogenic defects in S8/*Fis1* mice. Note the mitochondrial accumulation in spermatids before multinucleation. Note that spermatids arrest before step 9, when spermatids normally elongate and remodel their cytoplasm to form compacted sperm cells.

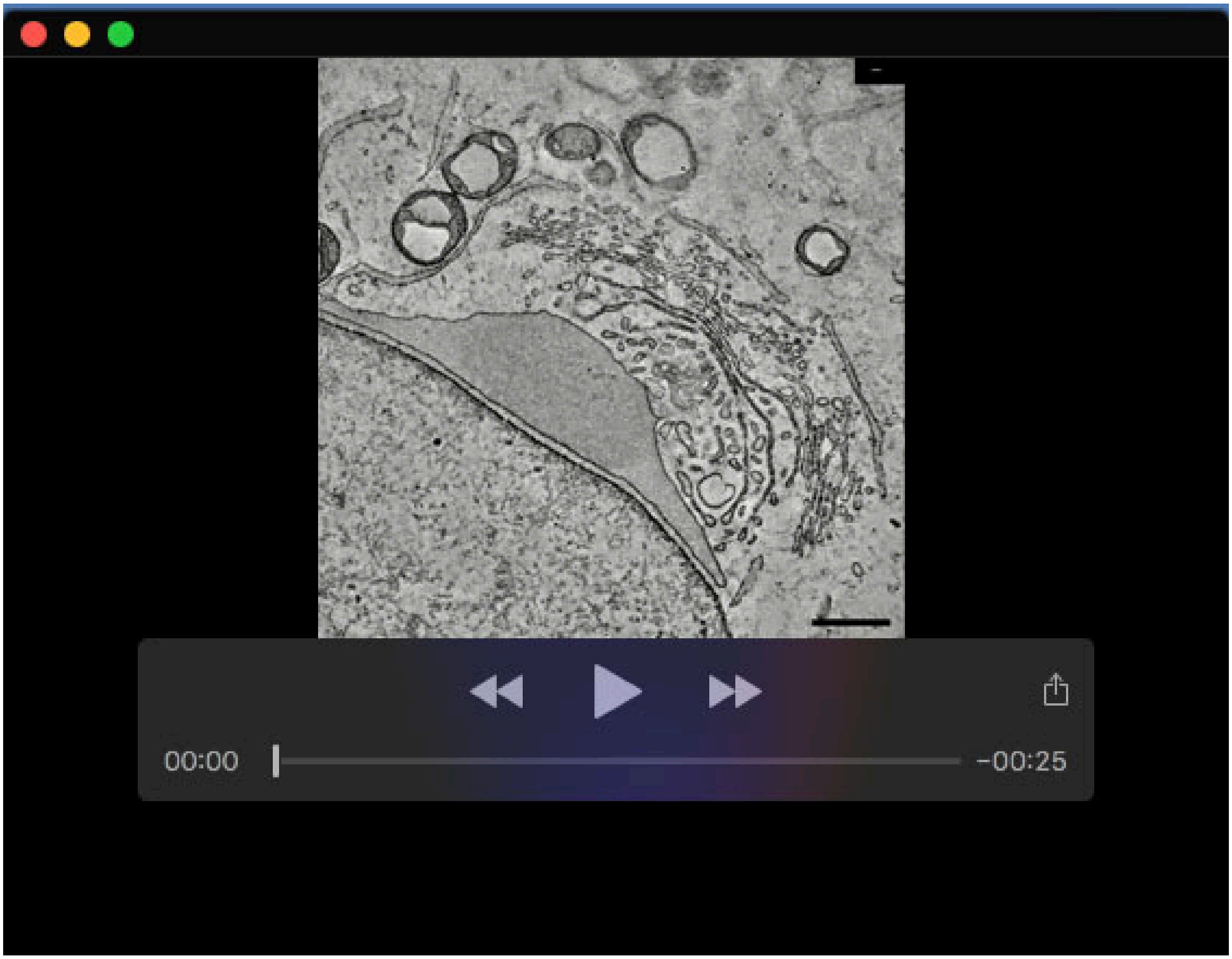

Movie 1. Related to Fig. 4C

Movie of P32 S8/Control spermatid showing an entire trans Golgi cisterna fused to the acrosome. Scale bars, 500 nm.

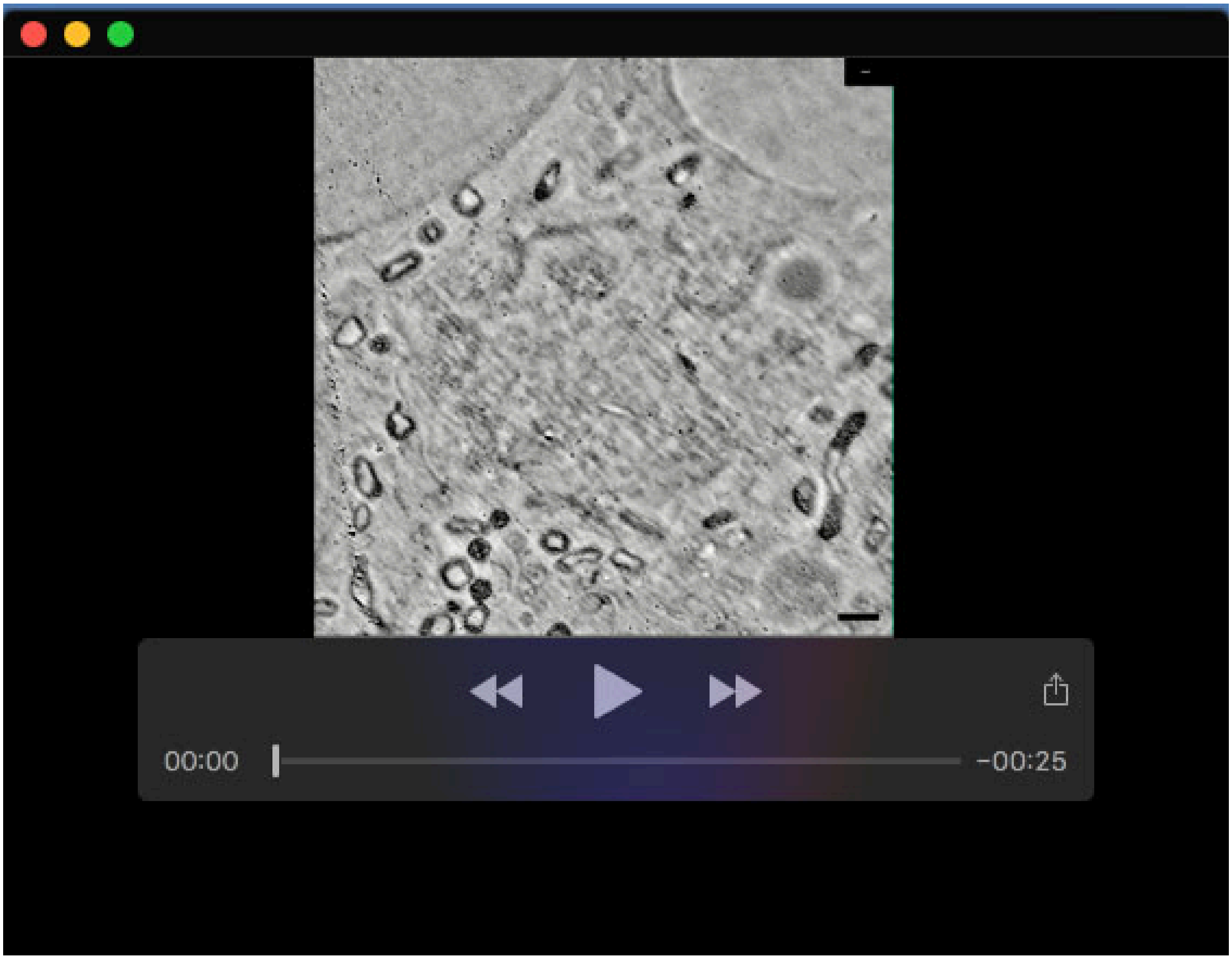

Movie 2. Related to Fig. 4C

Movie of a P32 S8/*Fis1* giant cell showing a proacrosomal granule and aberrant Golgi vesiculation. Scale bars, 500 nm.

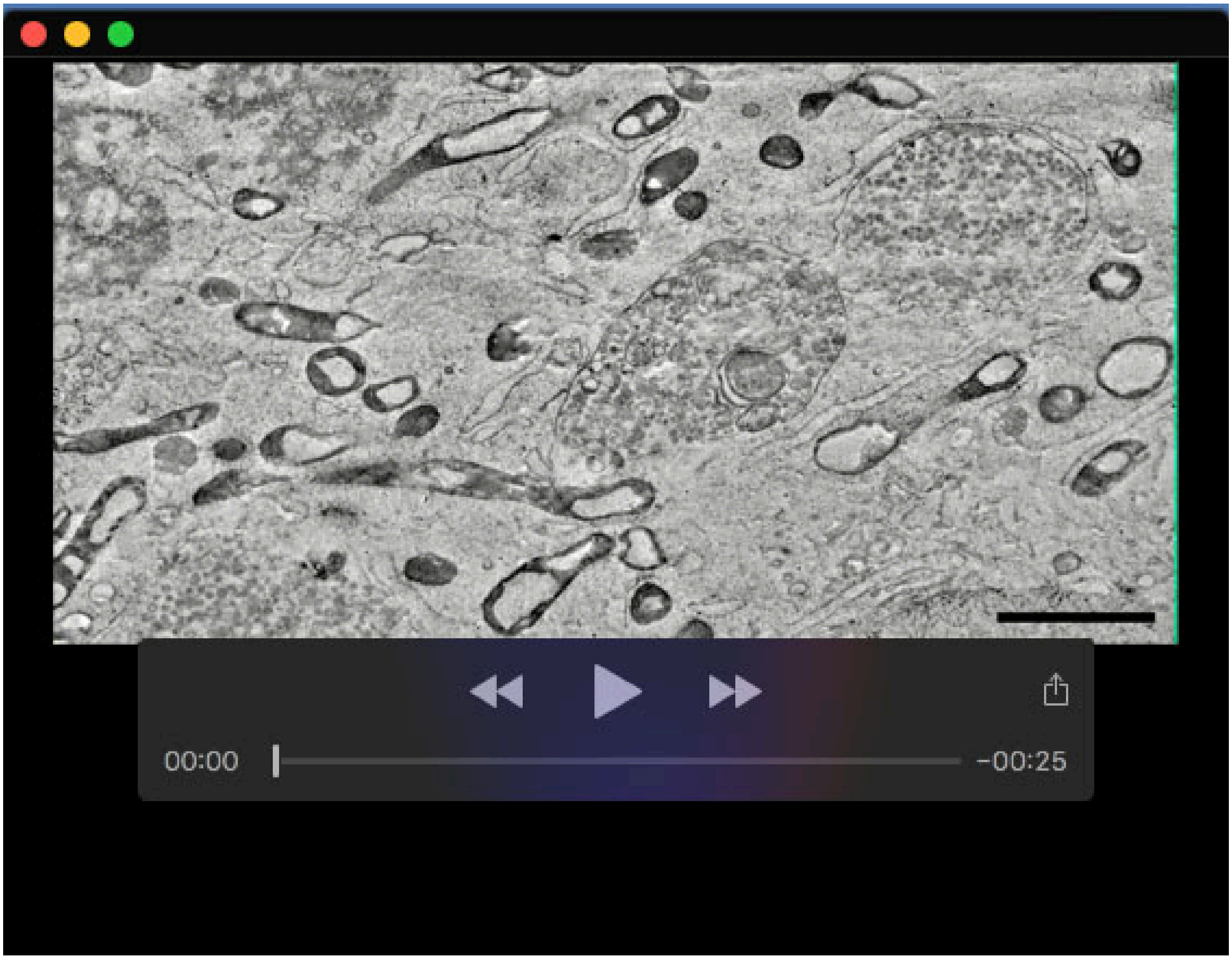

Movie 3. Related to Fig. 7B

Movie of a P32 S8/*Fis1* giant cell showing aberrant autophagic structures. Scale bar, 500 nm.

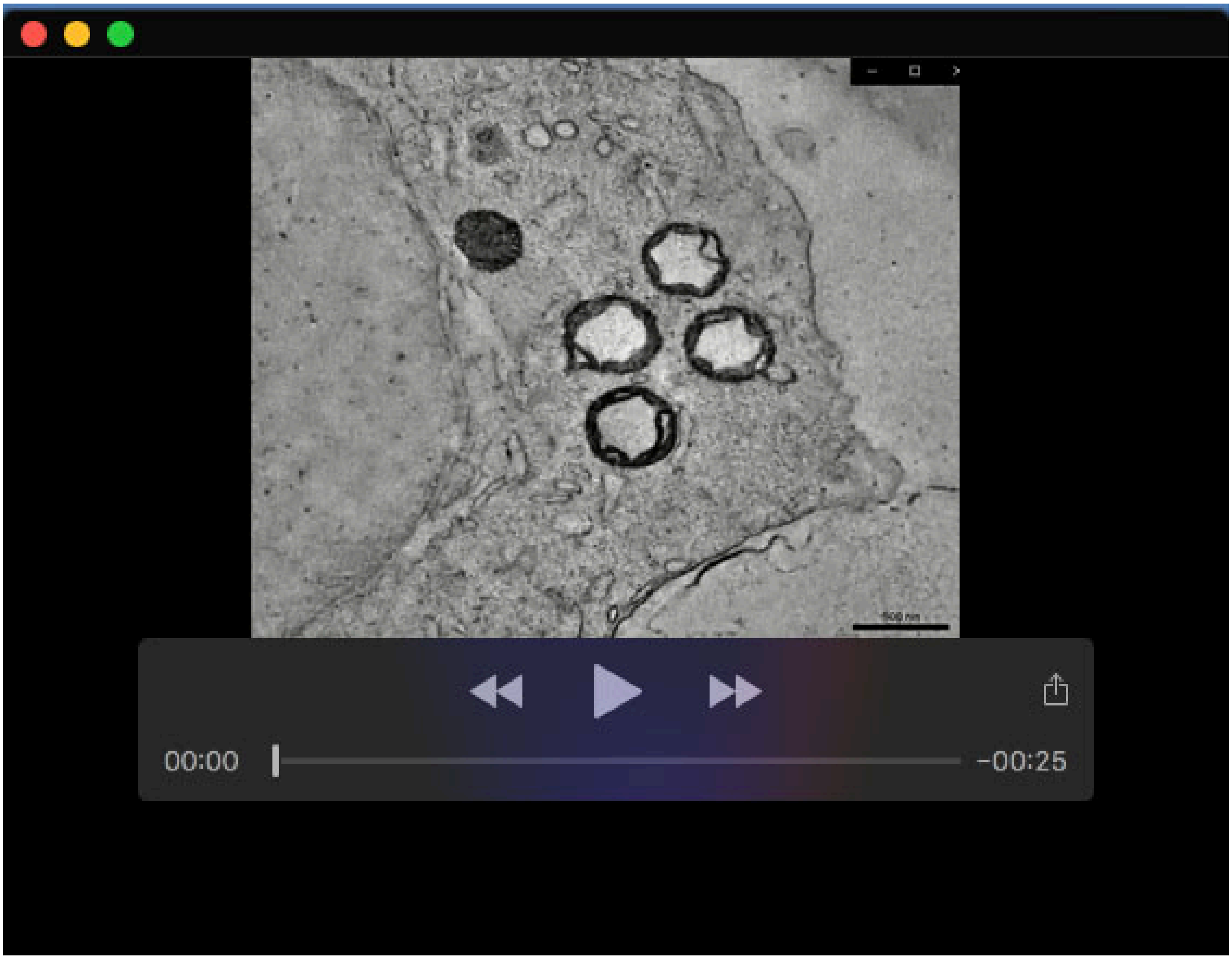

Movie 4. Related to Fig. 7B

Movie of a P32 control round spermatid showing small, spherical mitochondria. Scale bar, 500 nm.

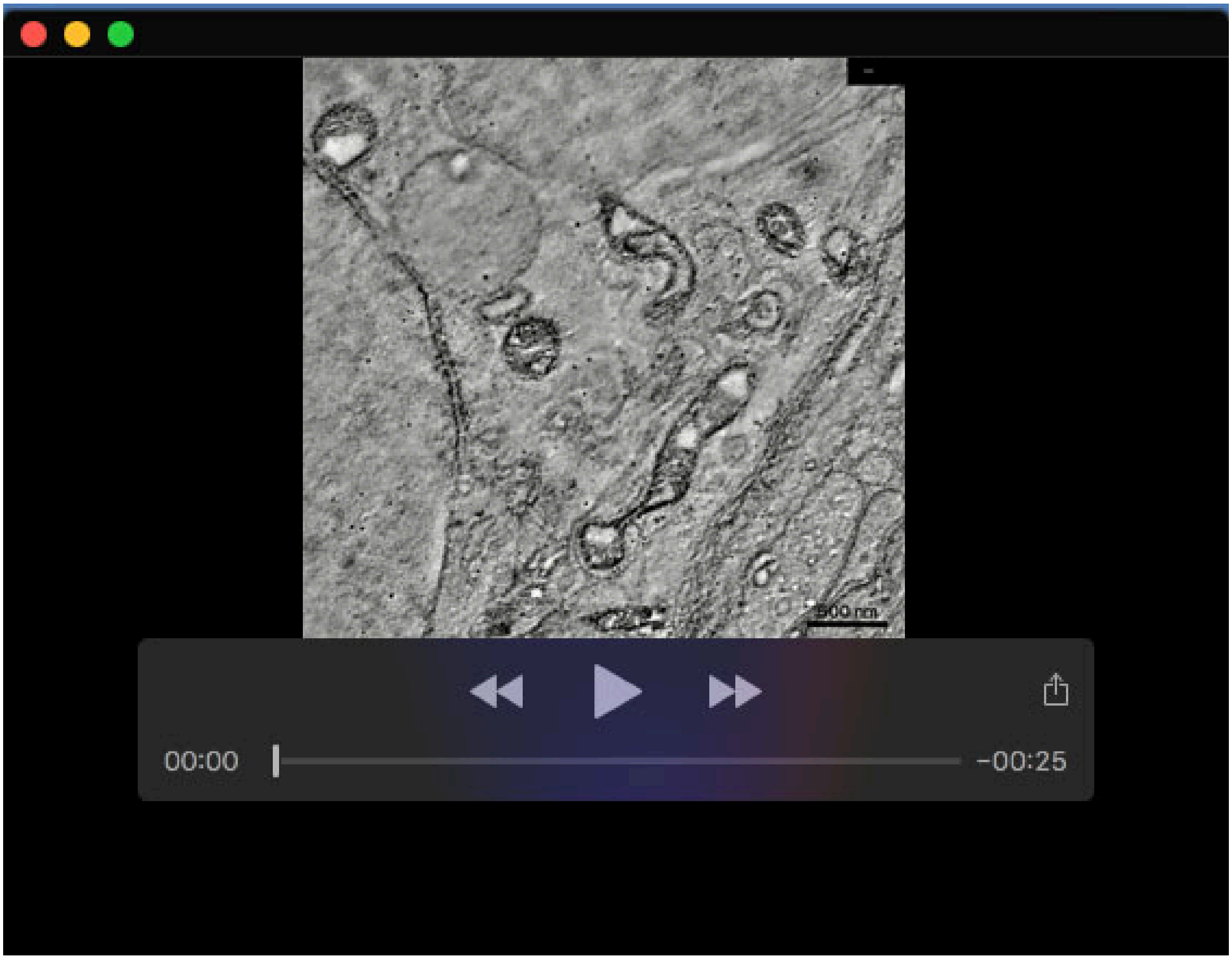

Movie 5. Related to Fig. 7B

Movie of elongated mitochondria with aberrant constrictions from a P24 S8/*Fis1* binucleated spermatid. Scale bars, 500 nm.

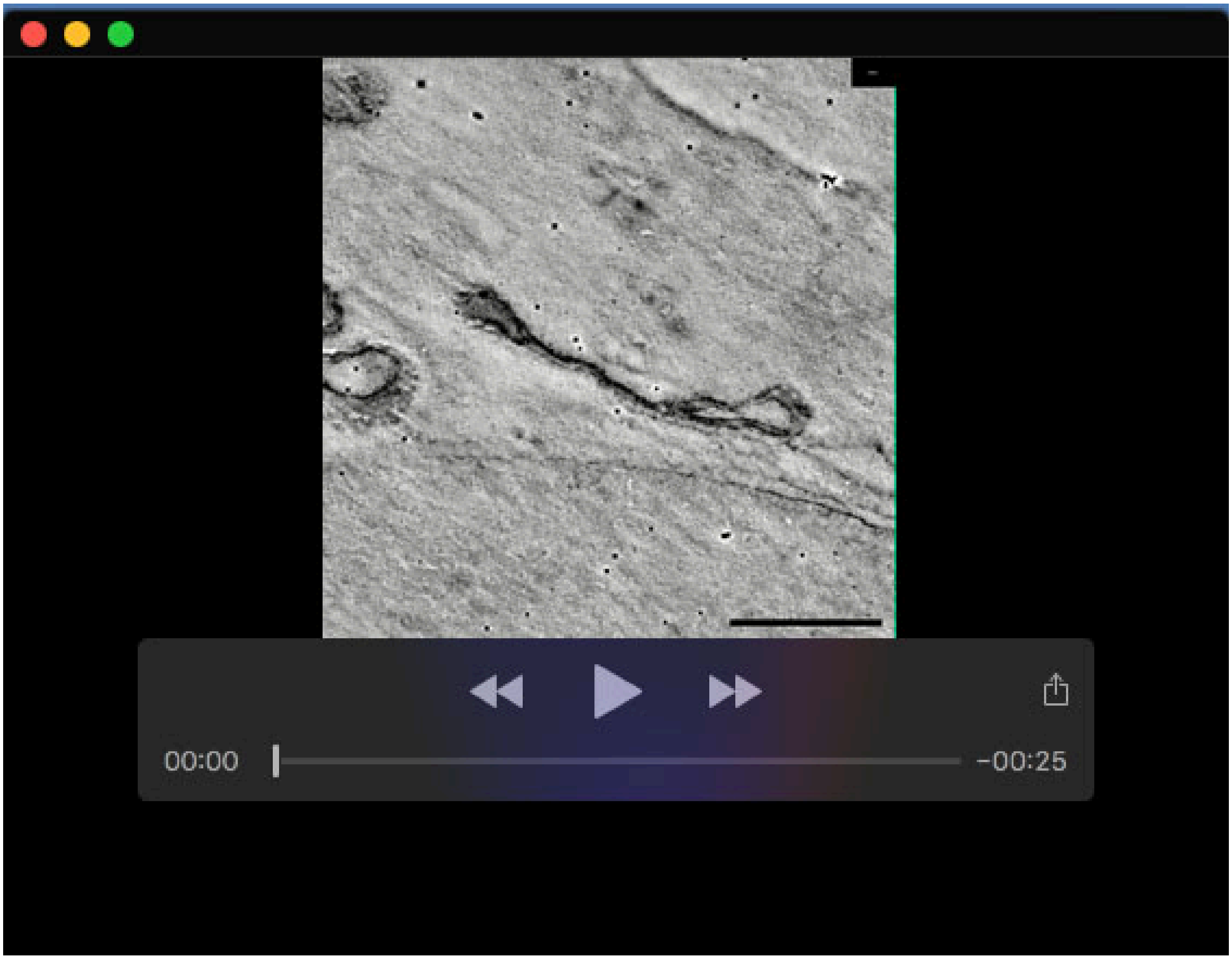

Movie 6. Related to Fig. 7B

Movie of an aberrantly constricted mitochondrion from a P32 S8/*Fis1* giant cell. Scale bar, 500 nm.

Development • Supplementary information

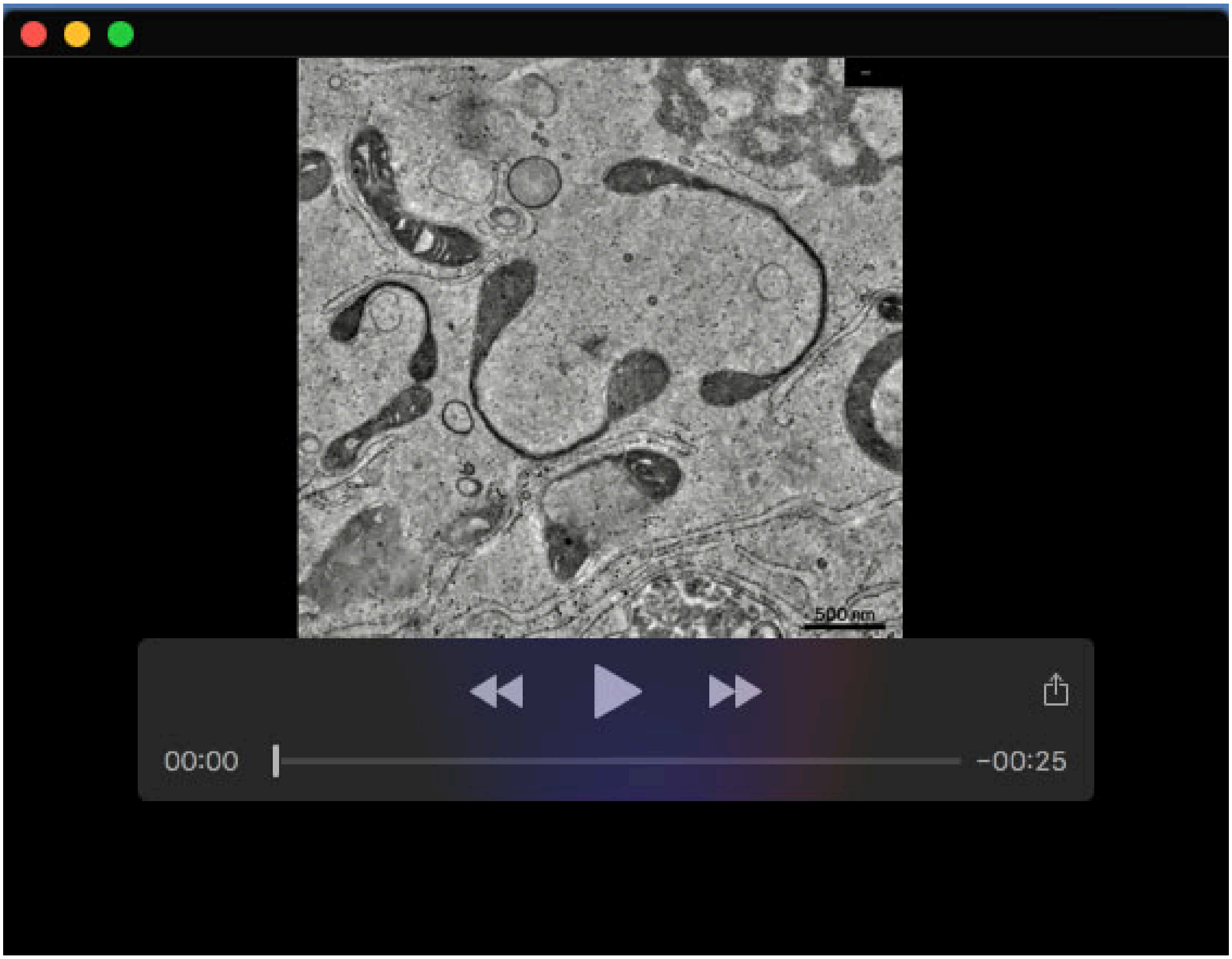

Movie 7. Related to Fig. 7B

Movie of aberrant dumbbell-shaped mitochondria from a P36 S8/*Fis1* giant cell. Scale bar, 500 nm.

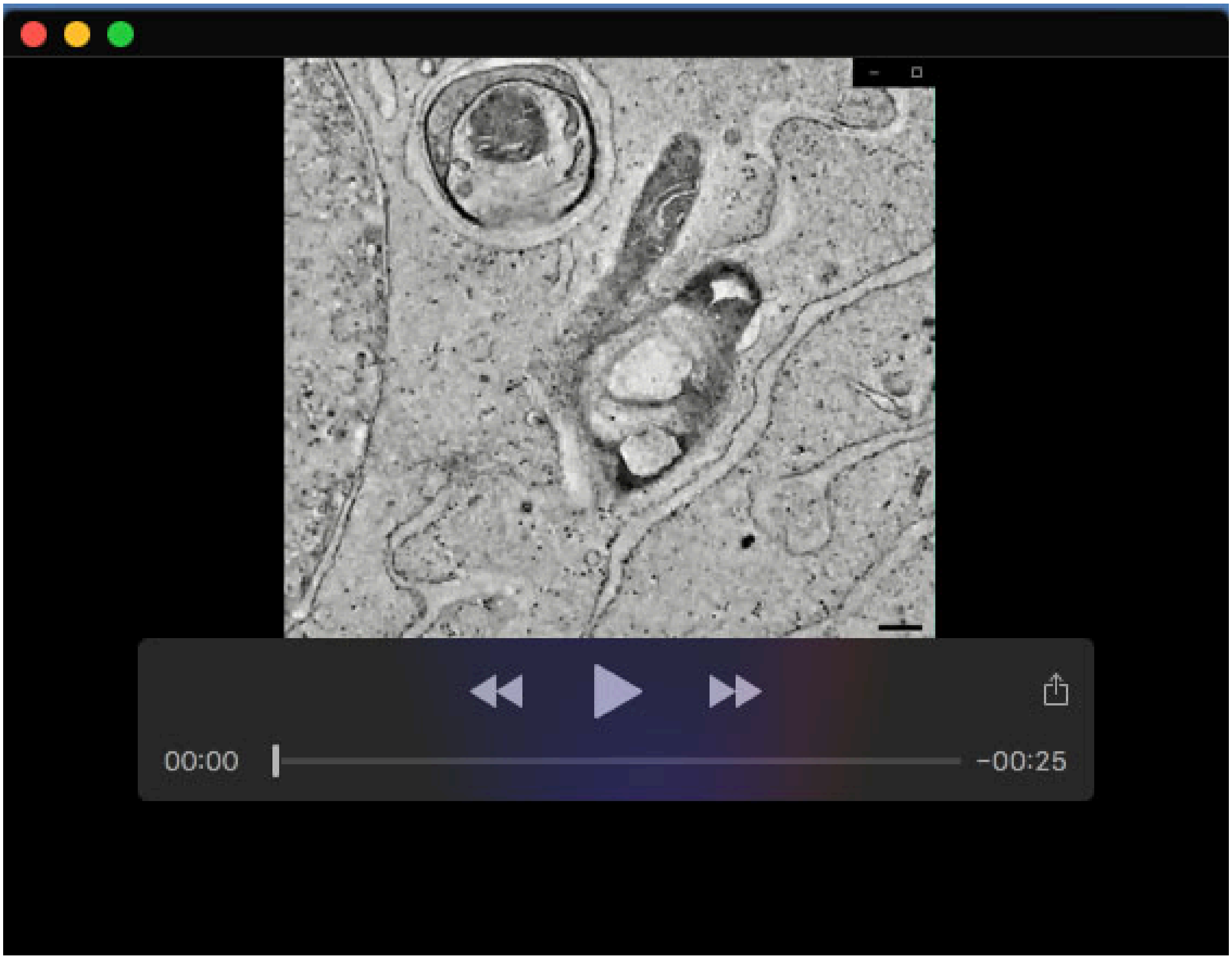

Movie 8. Related to Fig. 7C

Movie of a 3D reconstructed mitochondrion using serial section tomography from a P36 S8/*Fis1* giant cell. Scale bar, 500 nm.
