# Supplementary material for: Fis1 ablation in the male germline disrupts mitochondrial morphology and mitophagy, and arrests spermatid maturation
Source: Development. 2021 Aug 12;148(16):dev199686. doi: 10.1242/dev.199686 (PMC8380467; doi:10.1242/dev.199686)
Supplement: Reviewer comments [file develop-148-199686_review_history.pdf]

Fis1 ablation in the male germline disrupts mitochondrial morphology and mitophagy, and arrests spermatid maturation

Grigor Varuzhanyan, Mark Ladinsky, Shun-ichi Yamashita, Manabu Abe, Kenji Sakimura, Tomotake Kanki and David Chan

DOI: 10.1242/dev.199686

Editor: Swathi Arur

Review timeline

Original submission:	6 April 2021
Editorial decision:	13 May 2021
First revision received:	16 June 2021
Accepted:	13 July 2021

Original submission

First decision letter

MS ID#: DEVELOP/2021/199686

MS TITLE: Fis1 ablation in the male germline disrupts mitophagy and arrests spermatid maturation

AUTHORS: Grigor Varuzhanyan, Mark Ladinsky, Shun-ichi Yamashita, Manabu Abe, Kenji Sakimura, Tomotake Kanki, and David Chan

I have now received all the referees' reports on the above manuscript, and have reached a decision. The referees' comments are appended below, or you can access them online: please go to BenchPress and click on the 'Manuscripts with Decisions' queue in the Author Area.

As you will see, the referees express considerable interest and excitement in your work, but also provide recommendations for improving the manuscript. Specifically Reviewers 1 and 2 suggest significant textual changes to improve the manuscript, provide citations and add deeper context to the field in terms of the role of Fis1 and its associated phenotypes. In addition, reviewer 1 suggests a critical experiment in assaying the tubules in Figure 2E by more precise staging to better discern the time point at which the giant cells form. Similarly, reviewer 2's suggestion to assay the damaged mitochondria with another marker such as Mitotracker is useful. If you are able to revise the manuscript along the lines suggested, which may involve further experiments, I will be happy receive a revised version of the manuscript.

Please attend to all of the reviewers' comments and ensure that you clearly highlight all changes made in the revised manuscript. Please avoid using 'Tracked changes' in Word files as these are lost in PDF conversion. I should be grateful if you would also provide a point-by-point response detailing how you have dealt with the points raised by the reviewers in the 'Response to Reviewers' box. If you do not agree with any of their criticisms or suggestions please explain clearly why this is so.

Reviewer 1

Advance summary and potential significance to field

© 2021. Published by The Company of Biologists under the terms of the Creative Commons Attribution License (https://creativecommons.org/licenses/by/4.0/).

The is an interesting manuscript by Varuzhanyan, Ladinsky, Yamashita, Abe Sakimura, Kanki and Chan.
They report that germline-specific knockout of Fis1 leads to complete male sterility, with a block to progression at the round spermatid stage followed by giant cell (GC) formation, apoptosis and germ cell loss. Following this histological characterisation they go on to investigate mitochondria in the mutant model, focusing on mitochondria within the giant cell population. Fis1 is proposed to be mainly involved in stress-induced fission, so its involvement in spermatid development is interesting.

A significant caveat to the presentation of these data is that GC formation is a frequent and expected consequence of germ cell loss occurring at the round spermatid stage. This can be triggered in a range of mammalian species by many different stressors, both genetic and non-genetic.

* loss of cell adhesion molecules maintaining germ cell / Sertoli cell communication https://mcb.asm.org/content/mcb/26/9/3610.full.pdf
* reduction in p53 expression https://www.ncbi.nlm.nih.gov/pmc/articles/PMC47504/
* irradiation, hyperthermia https://www.sciencedirect.com/science/article/abs/pii/S0015028216385909 https://link.springer.com/article/10.1007/BF02144530
* pressure changes from efferent duct ligation https://pubmed.ncbi.nlm.nih.gov/3439852/
* steroid treatment https://onlinelibrary.wiley.com/doi/abs/10.1111/j.1439-0272.2009.00985.x
* treatment with thioglucose https://onlinelibrary.wiley.com/doi/abs/10.1111/j.1439-0272.1977.tb01680.x?sid=nlm%3Apubmed

GCs therefore are not a specific hallmark of any specific interruption to spermatogenesis but represent a common pathway of male germ cell death involving opening up of the cytoplasmic bridges between sister cells. It is not clear in this manuscript the stage at which the mitochondrial defects are arising or if the defect is due specifically to Fis1 regulation of mitochondria.

Comments for the author

In this study, the authors show in Fig1 and Fig2 that the GCs contain spermatids, and (as expected) that they are apoptotic, that they are enclosed by a single plasma membrane, and that they are eventually phagocytosed.
In figure 3 they show that GCs are γH2AX-positive, likely indicative of DNA damage, however, the lack of TUNEL staining shows that the DNA is not yet completely fragmented. γH2AX staining can sometimes precede TUNEL positivity, so it may be that these GCs are at different stages of degeneration. Alternatively, pan-nuclear γH2AX can sometimes occur independently of DNA double strand breaks in response to a variety of stresses including UV. They are appropriately circumspect about this observation in the discussion.

In figure 4 they address the question of when the phenotype initiates. GC formation can occur at multiple stages of spermatogenesis, and the specific stages of germ cell contained within each GC indicates the approximate developmental point at which progression is disrupted. In the current study, this is not very well characterised. In figure 4 they present a model in which GC formation occurs after step 5-8 of round spermatid development, however panel 4C clearly shows a step 2 spermatid (i.e. with the proacrosomal granule not yet in contact with the nucleus) within a GC. Their figure 4E also clearly shows step 2-4 spermatids within GCs.

It is possible that this may be variable between individuals or may vary between juvenile and adult animals – in particular the SP10 staining for juvenile animals in 4E does not seem consistent with that for adult animals in figure 4B. However, overall the majority of their figures show that the GCs have unorganised and diffuse staining for PAS-positive or SP10-positive material, suggesting that the acrosomes are still in very early developmental stages (step 1-2).
Moreover, if GC formation occurs only after steps 5-8 of development, it should be possible to identify tubules within the testis containing full layers of step 1-4 spermatids. (1) This is something that should be addressed by more precise staging of the tubules counted in figure 2E.

© 2021. Published by The Company of Biologists under the terms of the Creative Commons Attribution License (https://creativecommons.org/licenses/by/4.0/).

Overall, it seems from the data presented as though the onset of degenerative changes occurs substantially earlier than the authors propose, from the very beginning of round spermatid development. (2) They need to be more circumspect in their discussion of this, and more cautious about the model presented in figure 4F.

In figures 5-7 they address mitochondrial function and morphology within the GC population, and from this argue that the sterility phenotype is a consequence of disrupted mitochondrial events. I do not follow the argument here. GCs are a hallmark of a degenerative process and are already abnormal and either dead or dying. Although the changes in mitophagy (figures 5 & 6) and mitochondrial morphology (figure 7) are striking, it is possible that these could be a consequence of GC formation rather than a cause.

(3) Specific comment on the legend of figure 5A: Is this really a p24 mouse?
Mature cells with condensed nuclei are present.

(4) Figure 5b, the authors should normalise the mitochondrial fluorescence:nuclear count to another cell type on the image, e.g. spermatogonia mitochondrial fluorescence:nuclear count to give an internal control on each image.

(5) Also line 201-204, increased staining for COX and SDH does not necessarily indicate there is increased complex activity. The authors would need to test this directly to make this statement. The authors could rephrase this to restate as increased protein levels, not activity. Alternatively, they could assay directly for complex activity.

(6) To establish causality and thus mechanism, the authors should look at mitochondrial structure and function in any remaining spermatids that have not yet fused to form GCs. If there are none of these, this argues for an earlier onset of the phenotype than described.
Also, though this is outside the scope of the current study, future work could look at GCs in some other model of spermatid degeneration, to rule out the possibility that the mitochondrial abnormalities are a consequence rather than a cause of GC formation.
Finally, and significantly for this study, GC formation was observed in Pex13 germ cell knockout males with a defect in peroxisome biogenesis. The phenotype reported in the Pex13 knockout paper looks very similar to that seen in the current study. Since Fis1 also functions in peroxisome development, it is possible that this pathway, rather than the mitochondrial abnormalities shown may be the cause of GC formation and sterility in the Fis1 model. (7) The authors should stain for other peroxisomal markers including Pex13 to see if this is perturbed in these mutant testes.
https://www.nature.com/articles/s41598-019-45991-6

Reviewer 2

Advance summary and potential significance to field

Varuzhanyan et al. studied the role of Fis1 and its effects on mitophagy during spermatogenesis. To this end, the authors generated conditional Fis1 knockouts in the male germline of mice. They observed complete absence of spermatozoa in the epididymis of the knockout mice and increased numbers of apoptotic cells in testicular tubules. They also observed multinuclear giant cells in seminiferous tubules.
The expression of gamma-H2AX, which is normally restricted to meiotic divisions, persisted to what appears to be a later stage and was much stronger in the giant cells of the Fis1 mutants. Acrosomes formed during spermiogenesis, become fragmented and the fragments are dispersed in the Giant Cells.
Fis1 mutant spermatids have doubled their mitochondrial content, but additionally they accumulate aberrant autolysosomes as shown with LC3B and LAMP1 staining. Moreover, large amounts of p62 accumulate in the Giant cells suggesting that autophagic flux is blocked. Lastly, the authors observed peculiar dumbbell and bowl shaped mitochondria in the Giant Cells, suggesting that some other aspect controlling mitochondrial morphology is disrupted by the Fis1 mutations.

This manuscript describes a comprehensive and careful description of the phenomena affected by Fis1 mutations in the male germline.

© 2021. Published by The Company of Biologists under the terms of the Creative Commons Attribution License (https://creativecommons.org/licenses/by/4.0/).

Comments for the author

The observations are mostly in line with what could be expected based on previous Fis1 studies, but some questions stand out:

1. Are mitochondria in wildtype animals turned over to reduce mitochondrial mass or is the increase in the Fis1 mutant due to failure to remove damaged mitochondria? The increase in Ox-Phos complex activities suggests the former explanation, but some additional confirmation (if possible validation of membrane potential with MitoTracker) would make this a stronger case.

2. The accumulation of large autolysomes with LC3 in them, accumulation of p62 in the cytosol and the dispersal of Atg9A in Giant Cells suggest that Fis1 mutations are affecting multiple stages of mitophagy some of which may be indirect. The autolysosomes are acidic, since GFP is quenched, but proteolytic degradation seems to be stalled, so there might be problems delivering proteases to the lysosomes due to a general breakdown in vesicular transport. The authors could confirm this by testing whether delivery of proteases to the lysosome is compromised for example by staining for cathepsin.

3. It was interesting to note that Rab7, which is thought to be controlled by Fis1/TBC1D15 interactions during mitophagy, was observed surrounding phagosomes in Sertoli cells, but not in the mitophagosomes of Giant Cells. Can the authors comment on that?

Small points:

It would be helpful for the uninitiated to mention the stages of spermiogenesis at an earlier point in the manuscript and also to indicate how this staging relates to the timing of meiosis. It would also be helpful to explain what the significance of halting developmental arrest before step 9. Perhaps an extended schematic in the supplementary figures would do the trick.

The observed increase in apoptotic cells is unexpected, because Fis1 overexpression is also pro-apoptotic.
The authors should comment on this.

It would be interesting to contrast the staging results with those obtained previously with the Mff mutant.
In that study, the authors found progression to a different stage, they observed more constricted mitochondria and in contrast with the results here there was less complex IV activity. The dumbbell and bowl shaped mitochondria that were observed in Fis1 mutants could also result from a fission defect, even though the morphologies are different from those in Mff mutants.
Labeling in Fig. 1 for Dendra-green is DN, but this is not indicated in the figure legend.

<u>Reviewer 3</u>

Advance summary and potential significance to field

It is a very complete work that pinpoint the role of Fis1 along development of mice, from tissue morphology until molecular regulation.
I think this work is of interest for Development readers, because shed light on genetics and cellular players regulators of mitophagy in seminiferous tubules, and provide a link with the presence of giant multinucleated cells. This work permit to understand how Fis1 regulates mitophagy and how is linked to phenomenons like acrosome development.

Comments for the author

In the manuscript DEVELOP/2021/199686 “Fis1 ablation in the male germline disrupts mitophagy and arrests spermatid maturation”, the authors explored the role of Fis1 on mitochondrial dynamics and during spermatogenesis of mice. Authors focused in the mitophagic and autophagic control during spermatogenesis.

© 2021. Published by The Company of Biologists under the terms of the Creative Commons Attribution License (https://creativecommons.org/licenses/by/4.0/).

Questions arising, after initial characterization of S8/Fis1 males, were well concatenated and elegantly addressed. Results are sufficient, and are in line with to the objectives. Methods used in each stage of the investigation were appropriated and well interpreted.

First revision

Author response to reviewers' comments

Reviewer 1 Advance Summary and Potential Significance to Field:

The is an interesting manuscript by Varuzhanyan, Ladinsky, Yamashita, Abe, Sakimura, Kanki and Chan. They report that germline-specific knockout of Fis1 leads to complete male sterility, with a block to progression at the round spermatid stage followed by giant cell (GC) formation, apoptosis and germ cell loss. Following this histological characterisation they go on to investigate mitochondria in the mutant model, focusing on mitochondria within the giant cell population. Fis1 is proposed to be mainly involved in stress-induced fission, so its involvement in spermatid development is interesting.

A significant caveat to the presentation of these data is that GC formation is a frequent and expected consequence of germ cell loss occurring at the round spermatid stage. This can be triggered in a range of mammalian species by many different stressors, both genetic and non-genetic.

* loss of cell adhesion molecules maintaining germ cell / Sertoli cell communication https://mcb.asm.org/content/mcb/26/9/3610.full.pdf
* reduction in p53 expression https://www.ncbi.nlm.nih.gov/pmc/articles/PMC47504/
* irradiation, hyperthermia https://www.sciencedirect.com/science/article/abs/pii/S0015028216385909 https://link.springer.com/article/10.1007/BF02144530
* pressure changes from efferent duct ligation https://pubmed.ncbi.nlm.nih.gov/3439852/
* steroid treatment https://onlinelibrary.wiley.com/doi/abs/10.1111/j.1439-0272.2009.00985.x
* treatment with thioglucose https://onlinelibrary.wiley.com/doi/abs/10.1111/j.1439-0272.1977.tb01680.x?sid=nlm%3Apubmed

GCs therefore are not a specific hallmark of any specific interruption to spermatogenesis but represent a common pathway of male germ cell death involving opening up of the cytoplasmic bridges between sister cells. It is not clear in this manuscript the stage at which the mitochondrial defects are arising or if the defect is due specifically to Fis1 regulation of mitochondria.

Response: These are thoughtful comments. We appreciate the reviewer's point that giant cell formation may be a common response to a variety of defects in spermatids. In the revised Discussion, we note that giant cell formation occurs in response to several defects in spermatids and have included the reviewer's citations.

In P24 *Fis1* mutant mice, mitochondrial abnormalities precede giant cell formation (Fig. 5B, 7A, B), indicating that the mitochondrial abnormalities are not caused by giant cell formation. Furthermore, the mitochondrial abnormalities presented in our manuscript have not been reported in giant cells caused by other perturbations:
Efferent duct ligation:
https://pubmed.ncbi.nlm.nih.gov/3439852/

Loss of cell adhesion: https://journals.asm.org/doi/full/10.1128/MCB.26.9.3610-3624.2006#F9

Clinical Samples
https://onlinelibrary.wiley.com/doi/epdf/10.1111/j.1439-0272.1986.tb01729.x

© 2021. Published by The Company of Biologists under the terms of the Creative Commons Attribution License (https://creativecommons.org/licenses/by/4.0/).

Normal rabbits greater than 15 weeks old:
https://journals.sagepub.com/doi/10.1177/030098588602300211

Defect in peroxisome biogenesis: https://www.nature.com/articles/s41598-019-45991-6

These observations suggest that giant cell formation does not cause the defects we documented in the *Fis1* mutant giant cells. We have revised the Results and Discussion to better explain the timing of the mitochondrial defects in relation to giant cell formation. These points are further elaborated in response to the reviewer's related comments below.

Reviewer 1 Comments for the Author:
In this study, the authors show in Fig1 and Fig2 that the GCs contain spermatids, and (as expected) that they are apoptotic, that they are enclosed by a single plasma membrane, and that they are eventually phagocytosed.

In figure 3 they show that GCs are γH2AX-positive, likely indicative of DNA damage, however, the lack of TUNEL staining shows that the DNA is not yet completely fragmented. γH2AX staining can sometimes precede TUNEL positivity, so it may be that these GCs are at different stages of degeneration. Alternatively, pan-nuclear γH2AX can sometimes occur independently of DNA double strand breaks in response to a variety of stresses including UV. They are appropriately circumspect about this observation in the discussion.

In figure 4 they address the question of when the phenotype initiates. GC formation can occur at multiple stages of spermatogenesis, and the specific stages of germ cell contained within each GC indicates the approximate developmental point at which progression is disrupted. In the current study, this is not very well characterised. In figure 4 they present a model in which GC formation occurs after step 5-8 of round spermatid development, however, panel 4C clearly shows a step 2 spermatid (i.e. with the proacrosomal granule not yet in contact with the nucleus) within a GC. Their figure 4E also clearly shows step 2-4 spermatids within GCs.

It is possible that this may be variable between individuals or may vary between juvenile and adult animals – in particular the SP10 staining for juvenile animals in 4E does not seem consistent with that for adult animals in figure 4B. However, overall the majority of their figures show that the GCs have unorganised and diffuse staining for PAS-positive or SP10-positive material, suggesting that the acrosomes are still in very early developmental stages (step 1-2). Moreover, if GC formation occurs only after steps 5-8 of development, it should be possible to identify tubules within the testis containing full layers of step 1-4 spermatids. (1) This is something that should be addressed by more precise staging of the tubules counted in figure 2E. Overall, it seems from the data presented as though the onset of degenerative changes occurs substantially earlier than the authors propose, from the very beginning of round spermatid development. (2) They need to be more circumspect in their discussion of this, and more cautious about the model presented in figure 4F.

Response: We thank the reviewer for these excellent observations. In the revised manuscript, we modified our interpretation and presentation of the results regarding the timing of giant cell formation. The timing of giant cell formation was investigated by two analyses. In the first analysis, we determined the most advanced acrosome stages of spermatids enclosed in giant cells. In the second analysis, we looked at young mice whose spermatids were first forming to determine the most advanced spermatid stage before giant cell formation.

Analysis 1: To identify the stage of spermatids enclosed in giant cells, we looked at acrosome morphology using immunostaining and electron microscopy (Fig. 4B, C, D). By immunofluorescence, we found that the vast majority of acrosomes in GCs are small and fragmented, similar to acrosomes in steps 2-4 (Fig. 4B). Assuming that acrosome stages in giant cells reflect spermatid stages at the time of recruitment into giant cells, this observation suggests that giant cells largely arise from spermatids in steps 2-4. Our EM analysis shows that acrosomes in giant cells contain proacrosomal granules, which have a characteristic electron dense region near the center (Fig. 4C, D). We replaced the image in 4C to better illustrate this point and included a movie (Movie 2). The presence of proacrosomal granules in *Fis1* mutants further supports the model that early stage spermatids give rise to giant cells.

© 2021. Published by The Company of Biologists under the terms of the Creative Commons Attribution License (https://creativecommons.org/licenses/by/4.0/).

Analysis 2: We examined younger mice to characterize individual spermatids prior to giant cell formation (Fig. 4E). This analysis revealed that individual spermatids are indeed formed in *Fis1* mice. Most of the time, these individual spermatids had acrosomes corresponding to steps 2-4. Less frequently, we could also find individual spermatids with elongated acrosomes that resembled steps 5-8 (Fig. 4E). In mutant testes, we could find no individual spermatids beyond steps 5-8.

Based on these two analyses, we have updated our model to suggest that spermatids likely form giant cells predominantly between steps 2-4 of development (Fig. 4F). We also indicate a less common pathway wherein a smaller portion of spermatids reach steps 5-8 before forming giant cells.

In figures 5-7 they address mitochondrial function and morphology within the GC population, and from this argue that the sterility phenotype is a consequence of disrupted mitochondrial events. I do not follow the argument here. GCs are a hallmark of a degenerative process and are already abnormal and either dead or dying. Although the changes in mitophagy (figures 5 & 6) and mitochondrial morphology (figure 7) are striking, it is possible that these could be a consequence of GC formation rather than a cause.

Response: We understand the reviewers concern that the mitochondrial abnormalities may be the consequence, rather than the cause, of GC formation. However, as described above, the mitochondrial phenotypes we document in *Fis1* giant cells are not found in giant cells caused by other perturbations. In addition, we observed mitochondrial accumulation in individual spermatids (Fig. 5B) prior to their incorporation into giant cells. Furthermore, there is mitochondrial elongation and constriction at P24, when giant cell formation is just beginning (Fig. 7A,B,D and Movie 5). Along these lines, we have also included data showing a severe mitochondrial constriction in a P24 binucleated spermatid (Fig. 7A,B and Movie 5).

(3) Specific comment on the legend of figure 5A: Is this really a p24 mouse? Mature cells with condensed nuclei are present.

Response: Yes, these images were taken from a P24 mouse. The particular seminiferous tubule section shown in the WT panel was among the most advanced stage in this testis section.

(4) Figure 5b, the authors should normalise the mitochondrial fluorescence:nuclear count to another cell type on the image, e.g. spermatogonia mitochondrial fluorescence:nuclear count to give an internal control on each image.

Response: This is a good suggestion. We have updated Fig. 5C to include quantification of fluorescence intensity in spermatocytes. Note that the mitochondrial fluorescence intensity is increased in spermatids but not spermatocytes.

(5) Also line 201-204, increased staining for COX and SDH does not necessarily indicate there is increased complex activity. The authors would need to test this directly to make this statement. The authors could rephrase this to restate as increased protein levels, not activity. Alternatively, they could assay directly for complex activity.

Response: The COX/SDH enzyme stain is an established method for direct measurement of respiratory complex activity (not protein levels). The increased staining observed in mutant giant cells therefore indicates increased enzymatic activity. For clarity, we have pointed out in the main text that this could be due to increased activity of the protein complexes and/or increased content of mitochondria.

(6) To establish causality and thus mechanism, the authors should look at mitochondrial structure and function in any remaining spermatids that have not yet fused to form GCs. If there are none of these, this argues for an earlier onset of the phenotype than described. Also, though this is outside the scope of the current study, future work could look at GCs in some other model of spermatid degeneration, to rule out the possibility that the mitochondrial abnormalities are a consequence rather than a cause of GC formation.

© 2021. Published by The Company of Biologists under the terms of the Creative Commons Attribution License (https://creativecommons.org/licenses/by/4.0/).

Response: We have updated the text to clarify that mitochondrial abnormalities are already present at P24 in individual, binucleated, and early giant cells. For example, Fig. 5B shows mitochondrial accumulation in individual and binucleated spermatids. Furthermore, Fig. 7A, B, and D show mitochondrial constriction/elongation at P24. Finally, we have added EM tomograms of a binucleated spermatid, in which a severe mitochondrial constriction can be seen (Fig. 7A, B and Movie 5). Together, these observations indicate that early mitochondrial abnormalities precede giant cell formation.

Finally, and significantly for this study, GC formation was observed in Pex13 germ cell knockout males with a defect in peroxisome biogenesis. The phenotype reported in the Pex13 knockout paper looks very similar to that seen in the current study. Since Fis1 also functions in peroxisome development, it is possible that this pathway, rather than the mitochondrial abnormalities shown, may be the cause of GC formation and sterility in the Fis1 model. (7) The authors should stain for other peroxisomal markers including Pex13 to see if this is perturbed in these mutant testes. https://www.nature.com/articles/s41598-019-45991-6

Response: *Fis1* is primarily located on mitochondria but also has some localization to peroxisomes. We therefore agree with the reviewer that in addition to mitochondria, *Fis1* knockout may have an effect on peroxisomes. Although both the *Fis1* and *Pex13* knockouts have giant cells, it should be noted that the EM micrographs of Pex13 giant cells do not show any aberrant mitochondria. Thus the mitochondrial abnormalities we see in the *Fis1* knockout are unlikely due to a generic response to giant cell formation.

To examine peroxisomes in *Fis1* knockout testes, we stained testis sections with the peroxisome marker PEX14, which forms a complex with PEX13 (Fig. S5G). Notably, we do not find PEX14 staining in control round spermatids or in *Fis1* giant cells. We did observe a modest increase in staining in spermatocytes, and a more robust increase in spermatogonia at the tubule periphery. Thus, *Fis1* knockout may also be important for regulating peroxisome homeostasis during spermatogenesis.

Reviewer 2 Advance Summary and Potential Significance to Field: Varuzhanyan et al. studied the role of Fis1 and its effects on mitophagy during spermatogenesis. To this end, the authors generated conditional Fis1 knockouts in the male germline of mice. They observed complete absence of spermatozoa in the epididymis of the knockout mice and increased numbers of apoptotic cells in testicular tubules. They also observed multinuclear giant cells in seminiferous tubules. The expression of gamma-H2AX, which is normally restricted to meiotic divisions, persisted to what appears to be a later stage and was much stronger in the giant cells of the Fis1 mutants. Acrosomes, formed during spermiogenesis, become fragmented and the fragments are dispersed in the Giant Cells. Fis1 mutant spermatids have doubled their mitochondrial content, but additionally they accumulate aberrant autolysosomes as shown with LC3B and LAMP1 staining. Moreover, large amounts of p62 accumulate in the Giant cells suggesting that autophagic flux is blocked. Lastly, the authors observed peculiar dumbbell and bowl shaped mitochondria in the Giant Cells, suggesting that some other aspect controlling mitochondrial morphology is disrupted by the Fis1 mutations.

This manuscript describes a comprehensive and careful description of the phenomena affected by Fis1 mutations in the male germline.

Reviewer 2 Comments for the Author:
The observations are mostly in line with what could be expected based on previous Fis1 studies, but some questions stand out:

1. Are mitochondria in wildtype animals turned over to reduce mitochondrial mass or is the increase in the Fis1 mutant due to failure to remove damaged mitochondria? The increase in Ox-Phos complex activities suggests the former explanation, but some additional confirmation (if possible validation of membrane potential with MitoTracker) would make this a stronger case.

Response: We thank the reviewer for this important question. We agree that the increased respiratory chain complex activity suggests that mitophagy in spermatids acts to reduce

© 2021. Published by The Company of Biologists under the terms of the Creative Commons Attribution License (https://creativecommons.org/licenses/by/4.0/).

mitochondrial content, rather than to remove dysfunctional mitochondria.

We found that testes tissue could not be sectioned prior to fixation. Because MitoTracker incubation can only be performed with live cells, we dissociated testicular cells, stained with MitoTracker Red, and then immunostained against SP10 to identify round spermatids. For unknown reasons, WT and mutant round spermatids lacked MitoTracker staining (Fig. S5E). In contrast, other germ cell types had robust MitoTracker staining that colocalized with mito- Dendra. The MitoTracker staining in these other germ cell types was homogenous in S8/*Fis1* germ cells, indicating intact and uniform membrane potential (Fig. S5F).

2. The accumulation of large autolysomes with LC3 in them, accumulation of p62 in the cytosol and the dispersal of Atg9A in Giant Cells suggest that Fis1 mutations are affecting multiple stages of mitophagy some of which may be indirect. The autolysosomes are acidic, since GFP is quenched, but proteolytic degradation seems to be stalled, so there might be problems delivering proteases to the lysosomes due to a general breakdown in vesicular transport. The authors could confirm this by testing whether delivery of proteases to the lysosome is compromised for example by staining for cathepsin.

Response: This is an interesting suggestion, but none of the cathepsins tested in the literature have localized to spermatid lysosomes. Cathepsin D and H have been occasionally shown to express in spermatids, but the localization was restricted to the acrosome, not lysosomes. Furthermore, there are conflicting reports about the presence of acrosomal cathepsin. The relevant citations are below:

No Cathepsin D in mouse male germ cells.
https://www.sciencedirect.com/science/article/pii/S0065128112001407

No Cathepsin D in human male germ cells
https://pubmed.ncbi.nlm.nih.gov/21868745/
No Cathepsin B or D in rat male germ cells, with the exception of Cathepsin D on the acrosome.
https://pubmed.ncbi.nlm.nih.gov/7730593/

No Cathepsin B, D, or L in acrosome. Cathepsin H had localization to the acrosome, but this was only visible by immunoelectron microscopy, not immunofluorescence/light microscopy.
https://pubmed.ncbi.nlm.nih.gov/12838426/
No Cathepsin D in human male germ cells, with the exception of the acrosome.
https://onlinelibrary.wiley.com/doi/epdf/10.2164/jandrol.111.014639

3. It was interesting to note that Rab7, which is thought to be controlled by Fis1/TBC1D15 interactions during mitophagy, was observed surrounding phagosomes in Sertoli cells, but not in the mitophagosomes of Giant Cells. Can the authors comment on that?

Response: This is a good observation. We also specifically looked for RAB7A localization within the mitochondria of spermatids and found it notably absent. It is possible that another RAB, besides RAB7A, functions during mitophagy in spermatids, but more work will be necessary to confirm this hypothesis.

Small points:

It would be helpful for the uninitiated to mention the stages of spermiogenesis at an earlier point in the manuscript and also to indicate how this staging relates to the timing of meiosis. It would also be helpful to explain what the significance of halting developmental arrest before step 9. Perhaps an extended schematic in the supplementary figures would do the trick.

Response: Per the reviewers request, we defined the stages of spermiogenesis in the Introduction.

© 2021. Published by The Company of Biologists under the terms of the Creative Commons Attribution License (https://creativecommons.org/licenses/by/4.0/).

It is unclear why spermatid development arrests before Step 9, but this is when the spermatids begin to elongate and remodel their cytoplasm. It has been shown that autophagy (*Atg7*) is required for cytoplasmic remodeling during spermatid elongation. The blocks in mitophagy and perhaps general autophagy caused by *Fis1* KO may similarly prevent cytoplasmic remodeling. We have added a schematic to illustrate this point (Fig. S6).

The observed increase in apoptotic cells is unexpected, because Fis1 overexpression is also pro-apoptotic.
The authors should comment on this.

Response: We have included additional comments on the role of *Fis1* in apoptosis in the Results section.

It would be interesting to contrast the staging results with those obtained previously with the Mff mutant. In that study, the authors found progression to a different stage, they observed more constricted mitochondria and in contrast with the results here there was less complex IV activity. The dumbbell and bowl shaped mitochondria that were observed in Fis1 mutants could also result from a fission defect, even though the morphologies are different from those in Mff mutants.

Response: This is a good suggestion. Mitochondria in *Mff* mutants fail to divide during spermiogenesis, resulting in elongated/constricted mitochondria that fail to wrap around the axoneme to form the mitochondrial sheath. *Mff* mutants do not exhibit spermatogenic arrest or form giant cells, but they produce fewer sperm. Furthermore, *Mff* germ cells do not exhibit any obvious change in mitochondrial activity, but their epididymal spermatozoa do have reduced complex IV activity. We agree with the reviewer that bowl shaped mitochondria in the *Fis1* mutants could arise from a fission defect. Indeed, *Fis1* mitochondria elongate/constrict before forming bowls (Fig. 7D). We included these points in the Discussion section. Finally, a recent report showed that mitochondrial fission mediated by *Mff* and *Fis1* are separated in space, with the latter mediating fission at the organelle periphery to promote mitophagy (Kleele et al., 2021, *Nature* 593, 435–439).

Labeling in Fig. 1 for Dendra-green is DN, but this is not indicated in the figure legend.

Response: We have updated the figure legend to include a definition of Dn.

Reviewer 3 Advance Summary and Potential Significance to Field:
It is a very complete work that pinpoint the role of Fis1 along development of mice, from tissue morphologyuntil molecular regulation.

I think this work is of interest for Development readers, because shed light on genetics and cellular players regulators of mitophagy in seminiferous tubules, and provide a link with the presence of giant multinucleated cells. This work permit to understand how Fis1 regulates mitophagy and how is linked to phenomenons like acrosome development.

Reviewer 3 Comments for the Author:
In the manuscript DEVELOP/2021/199686 “Fis1 ablation in the male germline disrupts mitophagy and arrests spermatid maturation”, the authors explored the role of Fis1 on mitochondrial dynamics and during spermatogenesis of mice. Authors focused in the mitophagic and autophagic control during spermatogenesis. Questions arising, after initial characterization of S8/Fis1 males, were well concatenated and elegantly addressed. Results are sufficient, and are in line with to the objectives. Methods used in each stage of the investigation were appropriated and well interpreted.

Response: We thank the reviewer for the favorable review.

© 2021. Published by The Company of Biologists under the terms of the Creative Commons Attribution License (https://creativecommons.org/licenses/by/4.0/).

Second decision letter

MS ID#: DEVELOP/2021/199686

MS TITLE: Fis1 ablation in the male germline disrupts mitochondrial morphology and mitophagy, and arrests spermatid maturation

AUTHORS: Grigor Varuzhanyan, Mark Ladinsky, Shun-ichi Yamashita, Manabu Abe, Kenji Sakimura, Tomotake Kanki, and David Chan

ARTICLE TYPE: Research Article

I am happy to tell you that your manuscript has been accepted for publication in Development, pending our standard ethics checks.

Reviewer 1

Advance summary and potential significance to field

The authors have addressed our comments. The rebuttal does a good job of establishing that the mitochondrial abnormalities in the Fis1 null mice are not seen in other models of germ cell deterioration and giant cell formation.
They've cleared up the question over the timing of the onset of the phenotype and it is now well-presented. For the Fis1 staining they now have an image-
internal control for the signal, so that result is now better supported, and it is clearer that the mitochondrial accumulation precedes giant cell formation.

Comments for the author

The authors have addressed our comments. The rebuttal does a good job of establishing that the mitochondrial abnormalities in the Fis1 null mice are not seen in other models of germ cell deterioration and giant cell formation.
They've cleared up the question over the timing of the onset of the phenotype and it is now well-presented. For the Fis1 staining they now have an image-internal control for the signal, so that result is now better supported, and it is clearer that the mitochondrial accumulation precedes giant cell formation. We endorse acceptance of this manuscript.

Reviewer 2

Advance summary and potential significance to field

See previous reviews.

Comments for the author

The authors did a good job addressing the comments from the first round of reviews. At this point, I have not further comments.

Reviewer 3

Advance summary and potential significance to field

Presence of giant cells in seminiferous epithelium are a hallmark of many experimental studies. Role ablation of Fis1 in the origin of GC, disturbing in mitochondria and the carefully staging of spermatid maturation (and alterations in acrosome), could serve as basis for future studies using it as markers of toxic or physiological effects.

© 2021. Published by The Company of Biologists under the terms of the Creative Commons Attribution License (https://creativecommons.org/licenses/by/4.0/).

Comments for the author

In the manuscript DEVELOP/2021/199686V2 “Fis1 ablation in the male germline disrupts mitophagy and arrests spermatid maturation”, the authors expanded their explanations, to the previous manuscript, until literature make it possible. Sufficient remarks were made to the figure legends and in Introduction to make more understandable the Results and Discussion.

© 2021. Published by The Company of Biologists under the terms of the Creative Commons Attribution License (https://creativecommons.org/licenses/by/4.0/).
